# Single-cell analysis of the peripheral immune landscape in Parkinson’s disease: insights into dendritic cell and CD4+ T-cell transcriptomics

**DOI:** 10.1038/s41531-026-01283-1

**Published:** 2026-02-11

**Authors:** Sarah Meglaj Bakrač, Katarina Mandić, Lidija Cvetko Krajinović, Željka Mačak Šafranko, Fran Borovečki, Anja Barešić, Antonela Blažeković

**Affiliations:** 1 Department for Functional Genomics, Centre for Translational and Clinical Research, University Hospital Centre Zagreb, University of Zagreb School of Medicine, Zagreb, Croatia; 2 Laboratory for Computational Biology and Translational Medicine, Division of Electronics, Ruđer Bošković Institute, Zagreb, Croatia; 3 Department of Translational Medicine and Immunology, University Hospital for Infectious Diseases ‘Dr. Fran Mihaljević’, Zagreb, Croatia; 4 Department of Neurology, University Hospital Centre Zagreb, University of Zagreb School of Medicine, Zagreb, Croatia; 5 Biomedical Research Center Salata–BIMIS, University of Zagreb School of Medicine, Zagreb, Croatia; 6 Department for Anatomy and Clinical Anatomy, University of Zagreb School of Medicine, Zagreb, Croatia

**Keywords:** Computational biology and bioinformatics, Immunology, Neurology, Neuroscience

## Abstract

Parkinson’s disease (PD) is characterised by α-synuclein aggregation, dopaminergic neuron loss and chronic neuroinflammation. Disruption of the blood-brain barrier enables immune cell infiltration, including dendritic cells (DCs) and CD4+ T-cells, contributing to disease progression. To explore peripheral immune mechanisms in PD, we isolated DCs and CD4+ T-cells from the blood of 17 PD patients and 10 controls using magnetic separation, followed by flow cytometry and single-cell RNA sequencing. Cell-type annotation identified CD4+ T-cell and DC subtypes, including rare DC3 cells. PD patients showed reduced circulating DCs, with no change in CD4+ T-cell levels. Differential gene expression and pathway analysis suggest CD4+ effector memory T-cells (TEMs) and cDC2s as important mediators of immune responses in PD, enriched for immune-related pathways including T-cell activation and antigen presentation. Our findings implicate specific immune subsets in PD-associated neuroinflammation, suggesting cDC2s and CD4^+^ TEMs as potential targets for immunomodulatory strategies.

## Introduction

Parkinson’s disease (PD) is the second most common neurodegenerative disorder, affecting more than six million people worldwide and presenting a significant public health challenge^1^. It is a progressive disorder mainly affecting individuals over 60, with incidence rates varying by geographical region, ethnicity and race^2^. Clinical manifestations of the disease are varied and include a range of motor symptoms such as bradykinesia, rigidity, tremor and postural instability. Additionally, non-motor symptoms, including impaired sense of smell, dementia, insomnia, constipation and depression, can arise and often occur years before the diagnosis^3^. PD is classified as early-onset and late-onset based on the age of symptom onset, with 50 years as the threshold for classification^4^. Notably, only 5–10% of PD cases are familial (monogenic), while the majority are idiopathic, with the exact cause remaining unknown^5^. The cause of PD is thought to be multifactorial, involving a combination of both genetic and non-genetic factors that contribute to the development of the disease. Previous studies indicate that environmental factors, oxidative stress and mitochondrial dysfunction may also influence the onset and progression of PD^6–8^. However, the exact mechanisms involved are still not fully understood. Over the past two decades, great importance has been given to the activation of immune response and neuroinflammation in PD pathogenesis^9^.

Neuropathologically, PD is characterised by the intraneuronal accumulation of α-synuclein (αSyn) protein aggregates, known as Lewy bodies, and the progressive degeneration of dopaminergic neurons in the substantia nigra pars compacta. Neuronal accumulation of αSyn is thought to trigger an inflammatory response in the brain, causing microglia to adopt a pro-inflammatory state. This disruption impacts neuronal homoeostasis and synaptic function. Williams et al. demonstrated that αSyn overexpression in the central nervous system (CNS) myeloid cells results in elevated MHC II expression and increased T-cell infiltration, which is associated with neurodegeneration^10^. Additionally, αSyn has been shown to activate inflammasomes, resulting in the production of pro-inflammatory mediators^11^. Prolonged inflammatory processes in the patient’s brain are linked to increased blood-brain barrier (BBB) permeability. This facilitates the infiltration of immune cells and accelerates the degeneration of dopaminergic neurons^12,13^.

Microglia, the resident myeloid cells in the CNS, function as the brain’s macrophages and are crucial mediators of neuroinflammation^14,15^, whereas DCs serve as antigen-presenting cells (APCs) regulating the immune responses and inflammation in the periphery. They play a crucial role in initiating the primary immune response by presenting antigens to T-cells. As part of the innate immune system, DCs deliver antigens to naïve T-cells through MHC II molecules. This process activates the adaptive immune response and serves as a ‘bridge’ between the innate and adaptive immune systems^16^. Studies have shown that immune cells, such as DCs and CD4+ T-cells, infiltrate the CNS of patients with PD, where they significantly influence the functional phenotype of microglia and thus regulate the advancement of the neurodegenerative process^16–19^. The infiltration of immune cells and microglial activation promotes a pathological immune response characterised by increased proinflammatory cytokine and reactive oxygen species production, further exacerbating neuronal loss. Given that the disease is still incurable, this complex interaction between immune cells and neuronal health highlights the potential for targeted immunomodulatory therapies that could alleviate the effects of neuroinflammation in PD.

In recent years, research in the field of genomics and transcriptomics has emerged as one of the key aspects in elucidating disease mechanisms^20,21^. The advancement of single-cell sequencing technology has enabled scientists to study diseases at a cellular level, allowing for the identification of distinct cell populations and analysis of transcriptional changes within these cells^22–24^. Such an approach enables the identification of specific gene expression patterns associated with neuroinflammation and immune response in neurodegenerative diseases. In PD, accumulating evidence indicates that peripheral immune dysregulation contributes to disease pathogenesis. T-cells from PD patients have been shown to recognise αSyn-derived peptides, suggesting antigen-specific immune activation^25^. Single-cell transcriptomic studies have revealed clonal expansion of cytotoxic CD8+ and CD4+ T-cells^26^, alterations in natural killer (NK) cell populations associated with disease severity^27^, and distinct transcriptional profiles in αSyn-reactive T-cells, including depletion of cytotoxic effector memory subsets^28^. Similar single-cell analyses in Alzheimer’s disease have identified expansion of effector T-cell subsets in blood and cerebrospinal fluid, along with stage-dependent changes in T-cell reactivity to amyloid-B, highlighting shared neuroimmune mechanisms across neurodegenerative disorders^29,30^. Together, these studies underscore the value of single-cell approaches in uncovering peripheral immune heterogeneity and its potential contribution to PD.

To enhance our understanding of the complex immune mechanisms involved in PD, this study aimed to identify key cell subsets and pathways driving the immune response through single-cell RNA sequencing (scRNA-seq). CD4+ T-cells and DCs were selected as cells of interest due to their central roles in coordinating immune activity and their ability to influence brain-resident immune cells, potentially contributing to PD-related neuroinflammation. Our findings reveal a significant enrichment of immune pathways in CD4+ TEM and cDC2 subsets, including T-cell activation and cytokine signalling. We believe these cell subsets might play a crucial role in mediating neuroinflammation in PD. Moreover, we identified shared pathways between these subsets, highlighting their interplay. While previous studies have investigated immune responses in PD, the specific contributions of CD4+ TEM and cDC2 subsets to PD pathology have not been extensively explored. Our results underscore the importance of advanced technologies, such as scRNA-seq, in unravelling the mechanisms underlying this disease.

## Results

### Single-cell sequencing dataset and cluster annotation

To gain a better understanding of the role of immune cells in the pathology of PD, we conducted scRNA-seq on CD4+ T-cells and pre-enriched DCs population isolated from PBMCs. These cells were obtained from whole blood samples of 17 PD patients and 10 healthy controls. A summary of the sample features is presented in Table 1.

**Table 1:** Sample features of subjects included in single-cell RNA sequencing

Features	CTRL	PD
*N*	10	17
Demographic data		
Female, *n*(%)	4 (40)	10 (59)
Median age at inclusion (range)	60 (48–81)	65.5 (50–86)
Median age at onset (range)	n/a	61 (39–69)
Early-onset PD (<50 years), *n*		4
Late-onset PD (50+ years), *n*		11
Onset unknown, *n*		2

*PD* Parkinson’s disease, *CTRL* control, *n/a* not applicable.

We initially started scRNA-seq targeting a total cell recovery of 10,000 cells per sample to maximise the cell yield. After observing suboptimal sequencing saturation in the first batch (Supplementary Data 1), we adjusted the protocol to target a total recovery of 5,00 cells to meet sequencing depth requirements. Due to the sequencer capacity, samples were sequenced in batches of four. For each sample, we combined 2500 cells of each cell fraction (CD4 and DC) and used them further in GEX library preparation. During the separation process, PD sample S13 yielded only the CD4+ T-cell fraction. For this sample, we included a total of 5000 cells from the CD4+ T-cell fraction in the scRNA-seq. To assess transcriptional variability over time, we collected a biological replicate sample from one patient a week apart (S28 and S30). Additionally, PD sample S29 was excluded for its poor sample quality and low confidence score across all cell type clusters (see ‘Data validation’ for details).

**Fig. 1:**
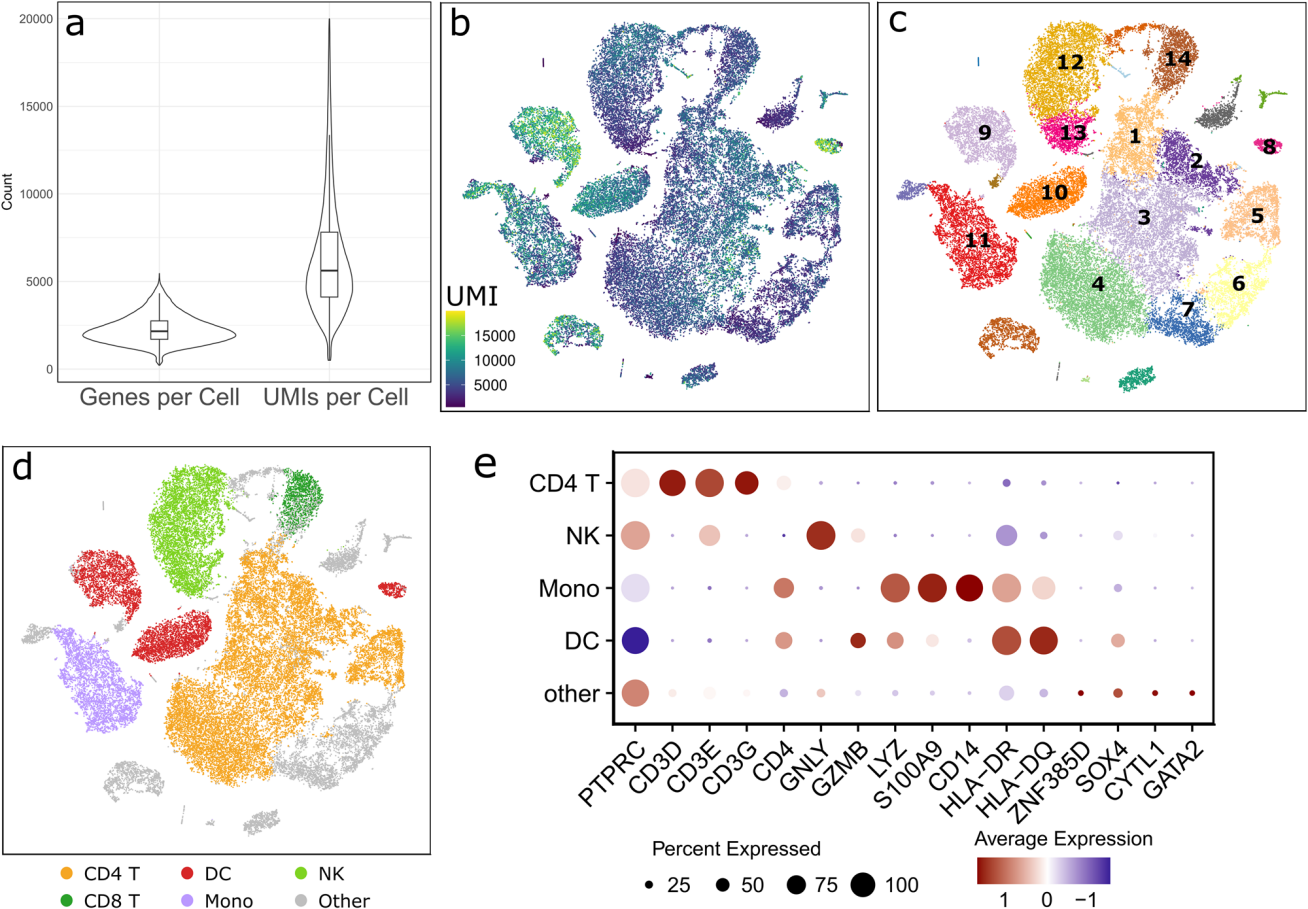
Single-cell sequencing data analysis. **a** Number of genes and UMIs found per cell. **b** tSNE plot of all cells coloured by the number of UMIs per cell, which distinguishes clusters with higher UMI content, identified as DCs (see later). **c** tSNE showing unsupervised SNN clustering in 27 clusters. Enumerated clusters correspond to major cell types identified in the data set. Clusters 1–5 are high confidence CD4+ T-cells, clusters 6 and 7 are low confidence CD4+ T-cells, clusters 8–10 are DC cells which also correspond to each of the three DC subtypes (cDC1, cDC2 and pDC), cluster 11 are monocytes, cluster 12 and 13 are NK cells and cluster 14 is identified as low confidence CD8 + T-cells. **d** tSNE showing final cell type annotation defined with reference-based annotation and SNN clustering. **e** Dot plot showing major cell types and marker genes selected based on previous literature findings^27,32,39,40^. The size of the dots represents the percentage of cells that express that marker. Colour shows the average expression of the marker gene.

After single-cell 5’ GEX sequencing, Cell Ranger processing, quality control and integration of all sample data, we obtained a total of 45,345 cells, containing 24,660 cells from PD samples and 20,685 cells from CTRL samples. The distribution of the number of genes and UMIs per cell after quality control is shown in Fig. 1a. To visualise our cells in two-dimensional space, we used the tSNE dimensionality reduction method. Figure 1b illustrates that specific cell clusters contain a higher number of UMIs per cell. The total number of reads and cells called is shown in Supplementary Data 1. We applied the RPCA method to integrate cells from all samples and identified 27 distinct cell clusters using SNN clustering (Fig. 1c).

### Identification of the main cell types in CD4+ T-cell and DC-enriched fractions

Cell type annotation was performed using a human PBMC reference dataset^31^. Cluster annotation was performed using a combination of SNN clustering and reference-based annotation. All obtained cells were originally annotated and sorted into a total of seven clusters: CD4+ T-cells, CD8+ T-cells, DCs, Monocytes, NK cells, other T-cells and ‘other’ (Supplementary Fig. 1a). Clusters ‘other T-cells’ and ‘other’ were merged into a single, dispersed cluster labelled ‘other’, referring to all cells that did not fall into one of the 5 specific types stated above. Furthermore, because of the low prediction scores (Supplementary Fig. 1b) and lack of CD3 complex gene expression, we have excluded a portion of CD4+ T-cells (Fig. 1c, clusters 6 and 7) called by reference-based annotation and included them into the cluster ‘other’ (Fig. 1d). Due to the low confidence score (Supplementary Fig. 1b), CD8+ T-cell cluster was also merged into the ‘other’ group. The final five clusters express common marker genes characteristic of their respective cell types, as shown in Fig. 1e. We demonstrate that some of the cells within the ‘other’ cluster express marker genes for granulocyte-monocyte progenitors (*ZNF385D*, *SOX4*, *CYTL1* and *GATA2*)^27^, potentially hindering their classification into one of the main cell clusters.

The results obtained from the scRNA-seq indicate the limitation of magnetic separation accuracy. Alongside the expected cell types of CD4+ T-cells and DCs, we obtained those that were unanticipated: monocytes, CD8 + T-cells and NK cells. Sample S13 clearly shows that a portion of monocytes and CD8 + T-cells remains within the CD4+ T-cells fraction, suggesting that the portion of NK cells present in our samples remains within the DC fraction (Supplementary Fig. 1c). Monocytes and NK cell clusters were identified as singular clusters and confirmed by assessment of specific marker gene expression. The markers used were *CD14*, *S100A9* and *CD68* for monocytes, whereas for the NK cells, we used *NKG7*, *GNLY* and *NCAM1* (Supplementary Fig. 1d)^32–35^.

Given that our samples are enriched for target cell populations (CD4+ T-cells and DCs), the resulting clusters of non-target cells do not accurately reflect the entire human immune phenotype. Consequently, these other cells were excluded from further comparison and differential gene expression analysis.

### Discovery of CD4+ T-cell subtypes

The tSNE embeddings identified CD4+ T-cells as a large singular cluster (Fig. 1d). All of these cells expressed the ‘signature’ marker genes encoding the CD3 complex subunits (CD3D, CD3E and CD3G) and CD4. Interestingly, most of our CD4+ T-cells express *CAMK4* and *BCL11B*, which are not common markers for CD4+ T-cells (Supplementary Fig. 1e).

**Fig. 2:**
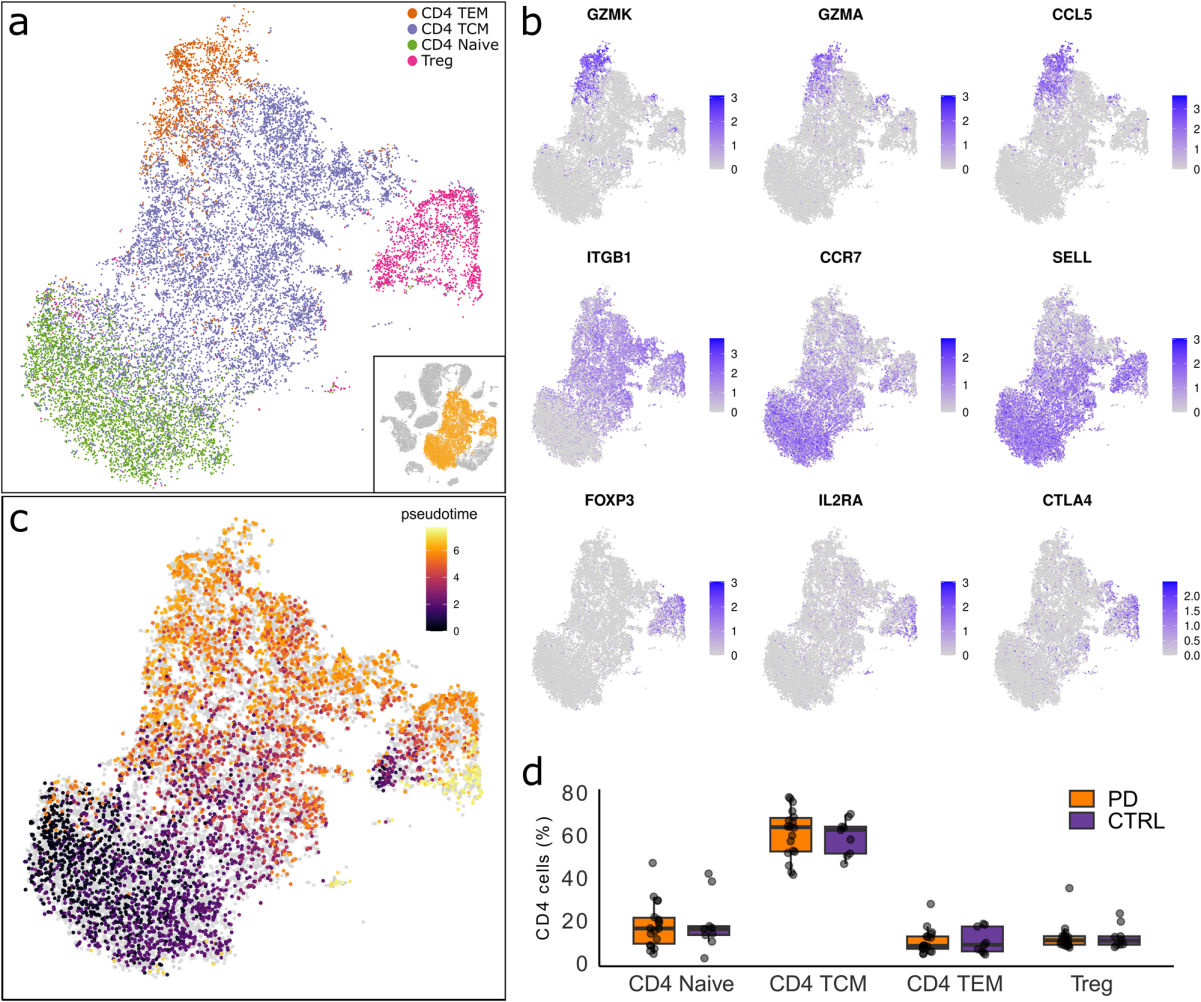
Single-cell analysis of CD4+ T-cell subtypes. **a** tSNE projection showing CD4+ T-cell subtype clusters. **b** Feature plots displaying the expression of CD4+ T-cell subtype marker genes, with higher expression levels indicated in purple. **c** tSNE visualisation illustrating a continuous pseudotime gradient across the CD4+ T-cell cluster. **d** Box plots comparing proportions of CD4+ T-cell subtypes among total CD4+ T-cell population between PD and CTRL samples. No significant differences were observed between the groups for any CD4+ T-cell subtype (Wilcoxon Rank Sum, *p* < 0.05).

Advanced annotation (i.e. reference annotation level 2) revealed four distinct CD4+ T-cell subtypes: CD4+ naïve T-cells, CD4+ central memory T-cells (TCMs), CD4+ effector memory T-cells (TEMs) and regulatory CD4+ T-cells (Tregs) (Fig. 2a). These subtype clusters were further confirmed by assessing the expression of specific marker genes shown in Fig. 2b. The markers correspond to the subtypes as follows: *GZMK*, *GZMA* and *CCL5* for CD4+ TEMs; *ITGB1* for CD4+ TCMs; *CCR7* and *SELL* for CD4+ naïve T-cells; and *FOXP3*, *IL2RA* and *CTLA4* for regulatory CD4+ T-cells. Selection of these marker genes was guided by a reference dataset and corroborated by findings in the literature^36–38^. To further characterise CD4+ T-cell subtypes we performed a pseudotime analysis, ordering cells along a learned trajectory based on their gene expression. As expected, the resulting pseudotime aligns with the differentiation states of CD4+ T-cell subtypes from naïve T-cells, to TCMs and TEMs (Fig. 2c and Supplementary Fig. 2a).

To investigate potential differences in CD4+ T-cell subtype proportions, we compared their proportions within a total number of CD4+ T-cells between the PD and CTRL groups using the Wilcoxon Rank Sum test (Fig. 2d). However, no significant difference was found for any of the CD4+ T-cell subtypes between the two groups (Supplementary Table 1).

### Detection of dendritic cell subtypes, including the rare DC3 cell population

**Fig. 3:**
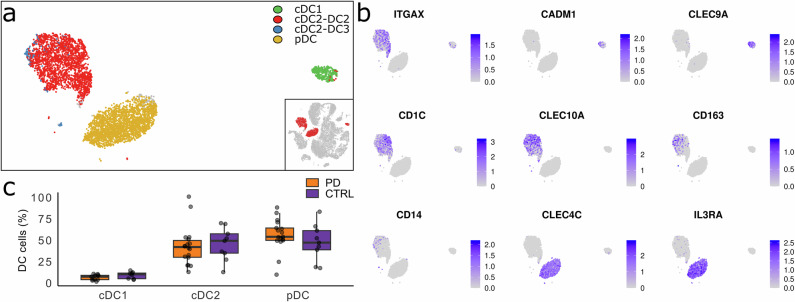
Single-cell analysis of dendritic cell (DC) subtypes. **a** tSNE projection showing DC subtype clusters. **b** Feature plots displaying the expression of DC subtype marker genes, with higher expression levels indicated in purple. **c** Box plots comparing proportions of DC subtypes among total DC population between PD and CTRL samples. No significant differences were observed between the groups for any DC subtype (Wilcoxon Rank Sum, *p* < 0.05).

SNN clustering identified three distinct clusters of DCs, which correspond well to DC subtypes identified using the reference-based annotation (Figs. 1d and 3a). The identified DC clusters correspond to three major DC subtypes: conventional (myeloid) DC1 (cDC1), conventional (myeloid) DC2 (cDC2) and plasmacytoid DC (pDC). The *HLA-DR* and *HLA-DQ* complexes are highly expressed in all three subtypes, while the *CD86* marker is more prevalent in the cDC subtypes. These subtype clusters were further confirmed by assessing the expression of specific marker genes shown in Fig. 3b. Marker gene *ITGAX* was used to highlight cDCs in general. Subtype-specific marker genes included *CADM1* and *CLEC9A* for cDC1s, *CDC1* and *CLEC10A* for cDC2s and *CLEC4C* and *IL3RA* for pDCs. Notably, we observed a small subset of cells expressing *CD163*, a marker gene uniquely associated with the DC3 subtype of cDC2s. Since DC3 cells are known to also express the monocyte marker gene *CD14*, its expression profile was shown in Fig. 3b to provide additional context^27,32,39,40^. The differentiation trajectory analysis did not show branching for DC subtypes (Supplementary Fig. 2b), which is consistent with their distinct developmental origins: cDCs originate from the myeloid lineage, whereas pDCs arise from a separate lymphoid progenitor.

Finally, we examined potential differences in DC subtype proportions by comparing these proportions within the total DC population between PD and CTRL groups (Fig. 3c). No significant differences were observed in any of the DC subtypes between the two groups by the Wilcoxon Rank Sum test (Supplementary Table 1).

### Flow cytometry shows altered proportions of dendritic cell subsets in PD patients

Flow cytometry was performed on PBMCs from PD patients and healthy controls. A slightly different cohort was used due to sample unavailability (Supplementary Table 2).

**Fig. 4:**
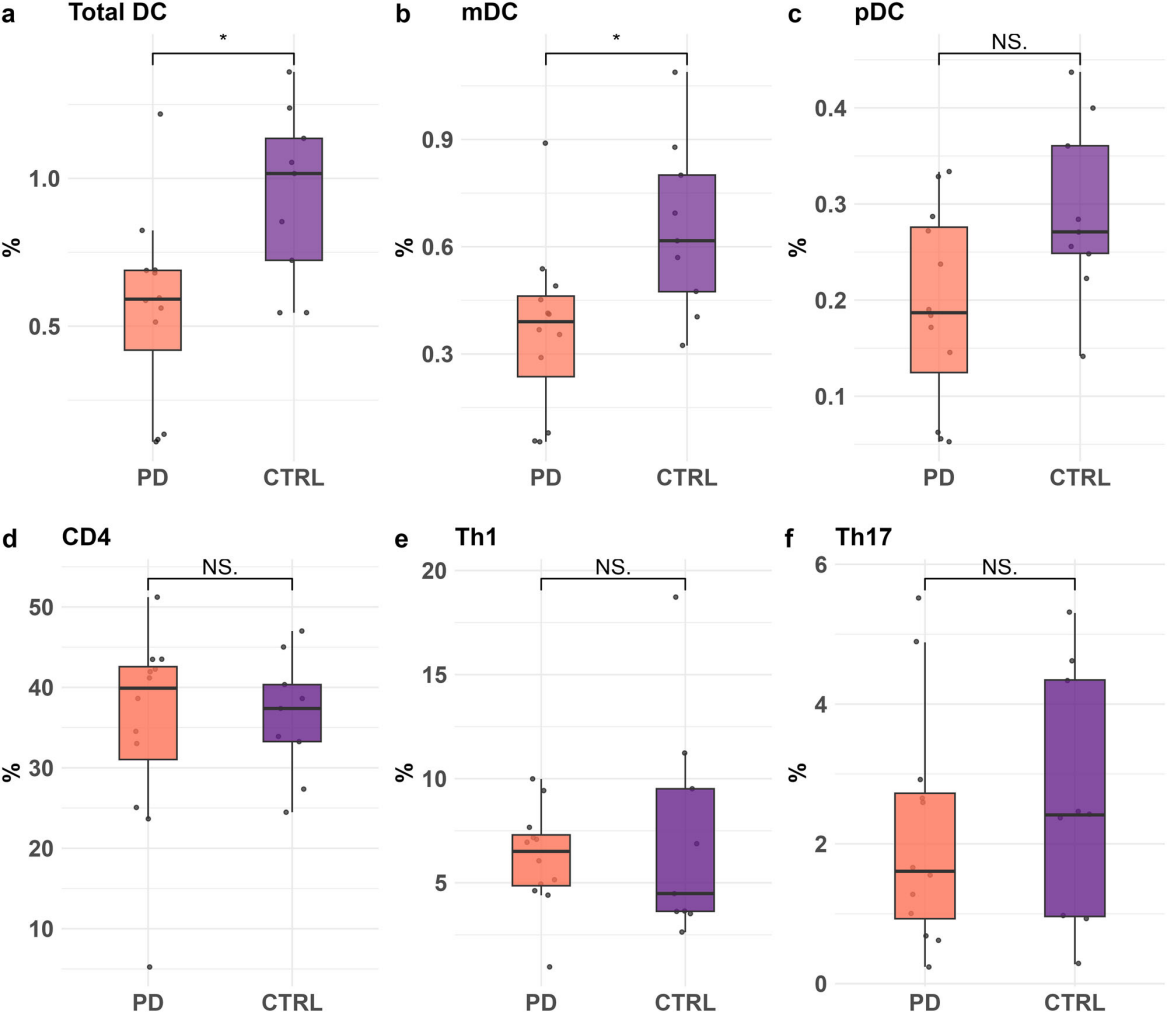
Flow cytometry analysis of dendritic cell (DC) and CD4+ T-cell subsets. Box plots represent the percentages of **a** total dendritic cells (DCs), **b** myeloid dendritic cells (mDCs), **c** plasmacytoid dendritic cells (pDCs), **d** CD4+ T-cells, **e** Th1 cells and **f** Th17 cells within peripheral blood mononuclear cells (PBMCs) from control (CTRL, purple, *n* = 9) and PD (orange, *n* = 12) groups. Data are expressed as frequencies of the cells in the leucocyte population for DCs and the lymphocyte population for CD4, Th1 and Th17. Statistical significance was determined using the Wilcoxon rank-sum test; *p* < 0.05 (*), and NS denotes non-significant differences. Each dot represents an individual donor.

Flow cytometry analysis revealed significant differences in DC subsets between the control (CTRL) and PD groups. The overall percentage of total DCs was significantly reduced in the PD group (*n* = 12), compared to CTRL (*n* = 9), *p* < 0.05, Fig. 4a. Further subset analysis demonstrated a significant decrease in mDCs, while the decrease in pDCs did not reach statistical significance in individuals with PD compared to controls (*p* < 0.05; Fig. 4b, c). In contrast, the frequency of CD4+ T-cells did not differ significantly between the two groups (Fig. 4d). Further analysis of CD4+ T-cell subsets showed no significant differences in the proportions of Th1 or Th17 cells between PD and CTRL individuals (Fig. 4e, f).

### Differential gene expression reveals immune pathways implicated in PD pathogenesis

**Fig. 5:**
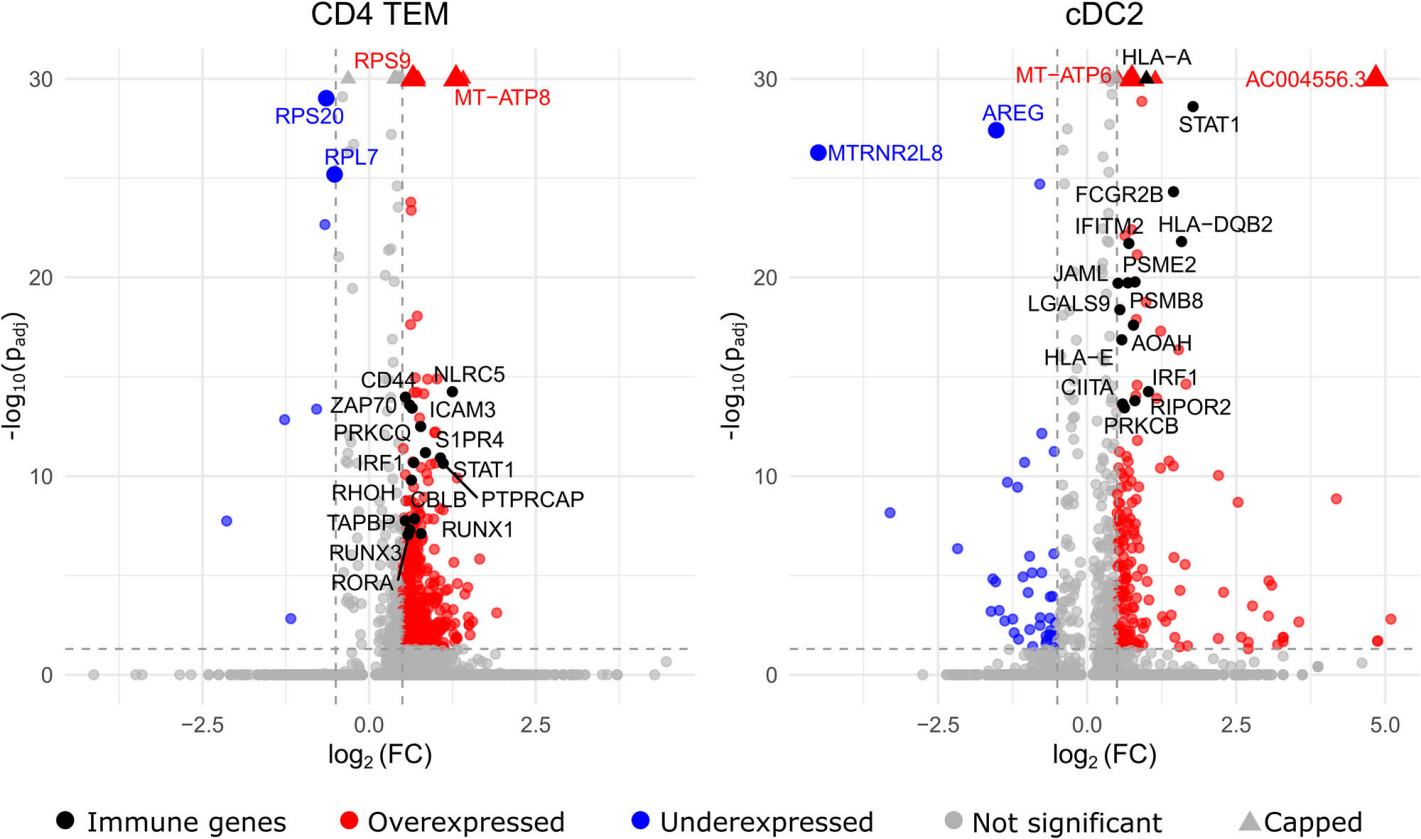
Volcano plots showing differentially expressed genes (DEGs) in CD4+ TEM (left) and cDC2 (right) cell subsets. Immune-related genes with adjusted *p* < 10^−10^ are labelled in black. Genes with very small *p* values (those capped at the upper limit of the *y*-axis) are predominantly mitochondrial and ribosomal protein genes. The complete list of DEGs is provided in Supplementary Data 2.

Using Seurat’s FindMarkers function, we identified DEGs between the PD and CTRL groups, focusing exclusively on four subtypes of CD4+ T-cells and three subtypes of DCs. CD4+ T-cells were analysed across four subclusters: naïve CD4+ T-cells, CD4+ TCMs, CD4+ TEMs and regulatory CD4+ T-cells. DCs were classified into three subtypes: cDC1s, cDC2s and pDCs. The total number of DEGs is summarised in Table 2 and differentially expressed genes for these subtypes can be found in Fig. 5 and Supplementary Data 2. The number of upregulated genes was considerably higher than the number of downregulated genes within all cell subtypes. Nonetheless, we included all differentially expressed genes in gene set enrichment analysis (GSEA).

**Table 2:** Numbers of differentially expressed genes in selected cell subtypes

Cell subtype	Total DEG	Upregulated	Downregulated
CD4 naïve T-cells	328	320	8
CD4 TCMs	1604	1593	11
CD4 TEMs	457	450	7
CD4 regulatory T-cells	210	207	3
cDC1s	18	16	2
cDC2s	230	190	40
pDCs	447	433	14

*DEG* differentially expressed genes, *TCMs* central memory CD4+ T-cells, *TEMs* effector memory CD4+ T-cells, *cDC* conventional dendritic cells, *pDCs* plasmacytoid dendritic cells.

To better understand the underlying functional differences detected DE levels might have on PD pathology, we conducted GSEA with the Gene Ontology biological processes (GO)^41^, Kyoto Encyclopaedia of Genes and Genomes (KEGG)^42^ and Reactome^43^ databases for each cell subtype’s DEGs. Because KEGG and Reactome terms were less informative, we performed further pathway analysis using only GO terms. For CD4+ T-cells, the DEGs of CD4+ naïve cells were enriched for only three terms. In contrast, DEGs of CD4+ TCM, CD4+ TEM and CD4+ Treg cells were enriched for 83, 180 and 46 terms, respectively. The full set of terms and results can be found in Supplementary Data 3. CD4+ TCM and Treg cells exhibited terms notably related to metabolic and biosynthetic processes, translation and gene expression, with very few terms associated with the immune system or immune processes. We further focused our analysis on CD4+ TEM cells. When activated, CD4+ TEMs are known to initiate polarisation of effector helper T-cells responsible for executing immune responses within the adaptive immune system. CD4+ TEM DEGs were enriched for a greater number of immune-related terms, including lymphocyte activation and differentiation, compared to other CD4+ T-cell subsets (Supplementary Data 3). In addition to immune-related terms, other notable enrichments included RNA metabolism and ribosomal subunit processes, potentially reflecting the involvement of ribosomal protein genes.

For DCs, DEGs of cDC1s were enriched for 11 pathways, with several terms related to antigen processing and presentation (Supplementary Data 3). In contrast, cDC2 DEGs were enriched for 129 pathways, largely involving processes related to immune response, such as antigen processing and presentation, lymphocyte activation, cytokine production and regulation of various biological and immune processes. Our analysis, therefore, concentrated on pathways essential to fundamental DC functions: antigen processing and presentation, lymphocyte (T-cell) activation and cytokine production and the cDC2 subtype, only. As for pDCs, only one enriched term was identified, i.e. ‘gene expression (transcription)’.

**Fig. 6:**
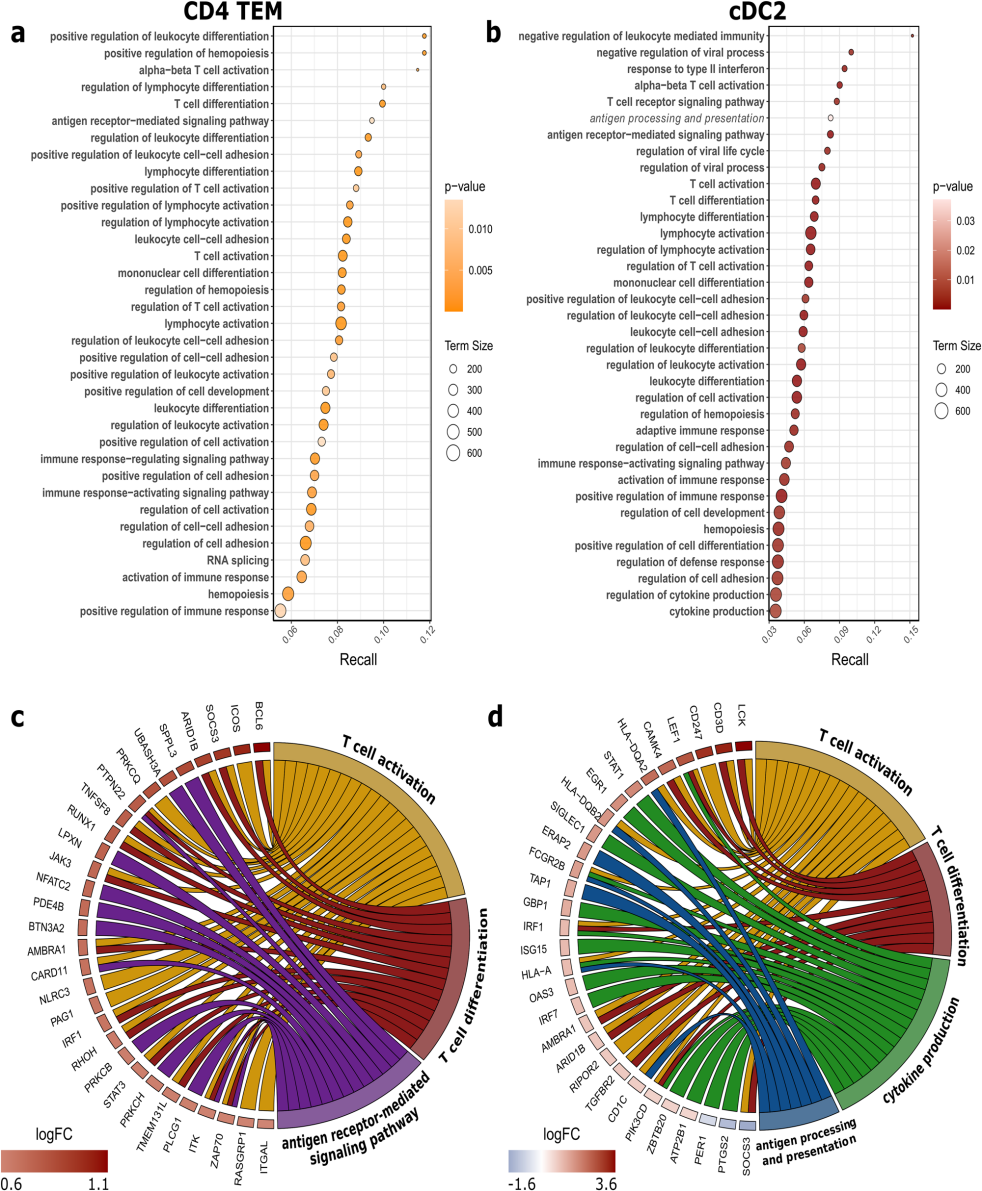
Pathway enrichment analysis results for CD4+ TEM and cDC2 cells. **a, b** Gene ontology pathways (terms) listed by recall for differentially expressed genes (DEGs) in the PD group compared to the CTRL group. The size of the circles indicates term size, and the dot colour represents *p* value. **c, d** Circos plots showing selected pathways and the top 30 DEGs, ranked by log fold-change (logFC) values. **a**, **c** correspond to CD4+ TEM cells, and **b**, **d** correspond to cDC2 cells.

We highlighted the top 30 GO terms for CD4+ TEM cells and cDC2s based on p-value, ranked them by recall values in Fig. 6a, b, and selected terms that best represented the primary functions of each cell type in Fig. 6c, d.

### Protein–protein interaction network analysis supports GSEA results

**Fig. 7:**
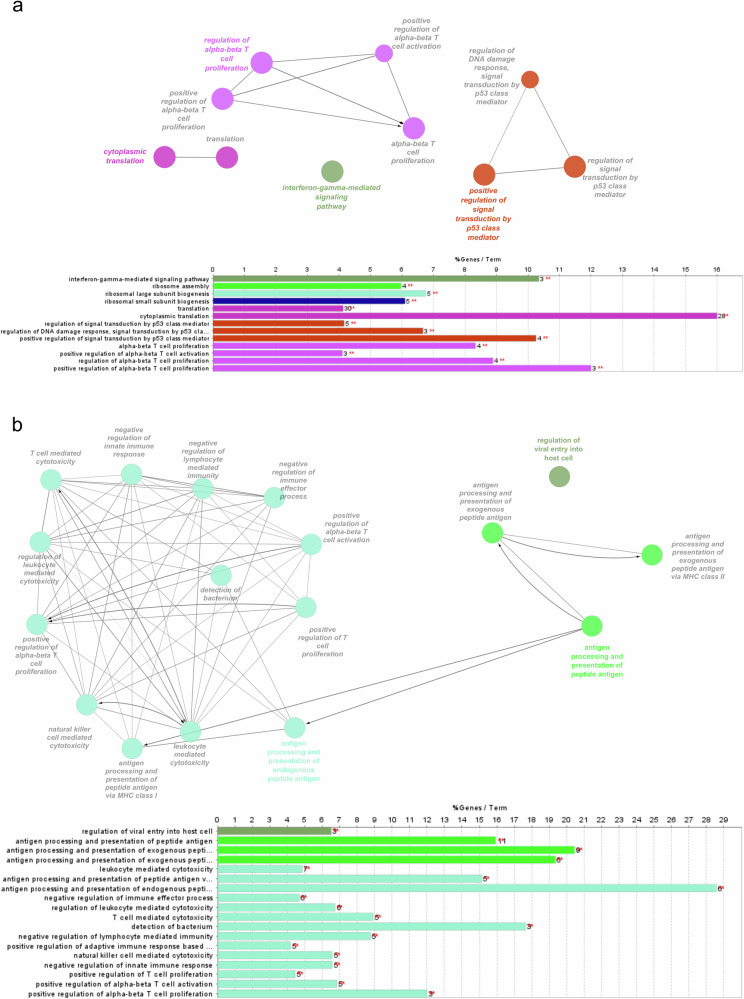
Functional clusters of protein-protein interaction (PPI) networks derived from differentially expressed genes (DEGs) of CD4 + TEM (**a**) and cDC2 (**b**) cells. In both **a**, **b** the upper sections illustrate functional clusters based on Gene Ontology, where the node colour represents different biological processes. The bottom sections indicate the number of DEGs associated with each term.

The protein–protein interaction (PPI) network was constructed based on the DEGs of CD4+ TEM cells and cDC2s. Figure 7 illustrates the significant functional clusters identified through the PPI network analysis using ClueGO. In CD4+ TEM cells, the most significant biological processes and pathways were linked to translation and alpha/beta T-cell proliferation, consistent with an activated T-cell phenotype. In contrast, the cDC2 functional clusters were enriched in processes related to antigen processing and presentation, in line with the previously reported pathway enrichment analysis results for CD4+ TEM and cDC2 cells. Additionally, enrichment was observed in biological processes such as T-cell activation and proliferation. Overall, the significant DEGs in the PPI network reveal two distinct functional clusters that correspond to CD4+ TEM and cDC2 cells, with minimal overlap as shown in Fig. 7. The only overlap observed is in T-cell activation pathways, which suggests potentially shared immune coordination between these cell types and corroborates the findings from the pathway enrichment analysis.

### Cell–cell communication analysis reveals enhanced CD4–dendritic cell signalling in PD

**Fig. 8:**
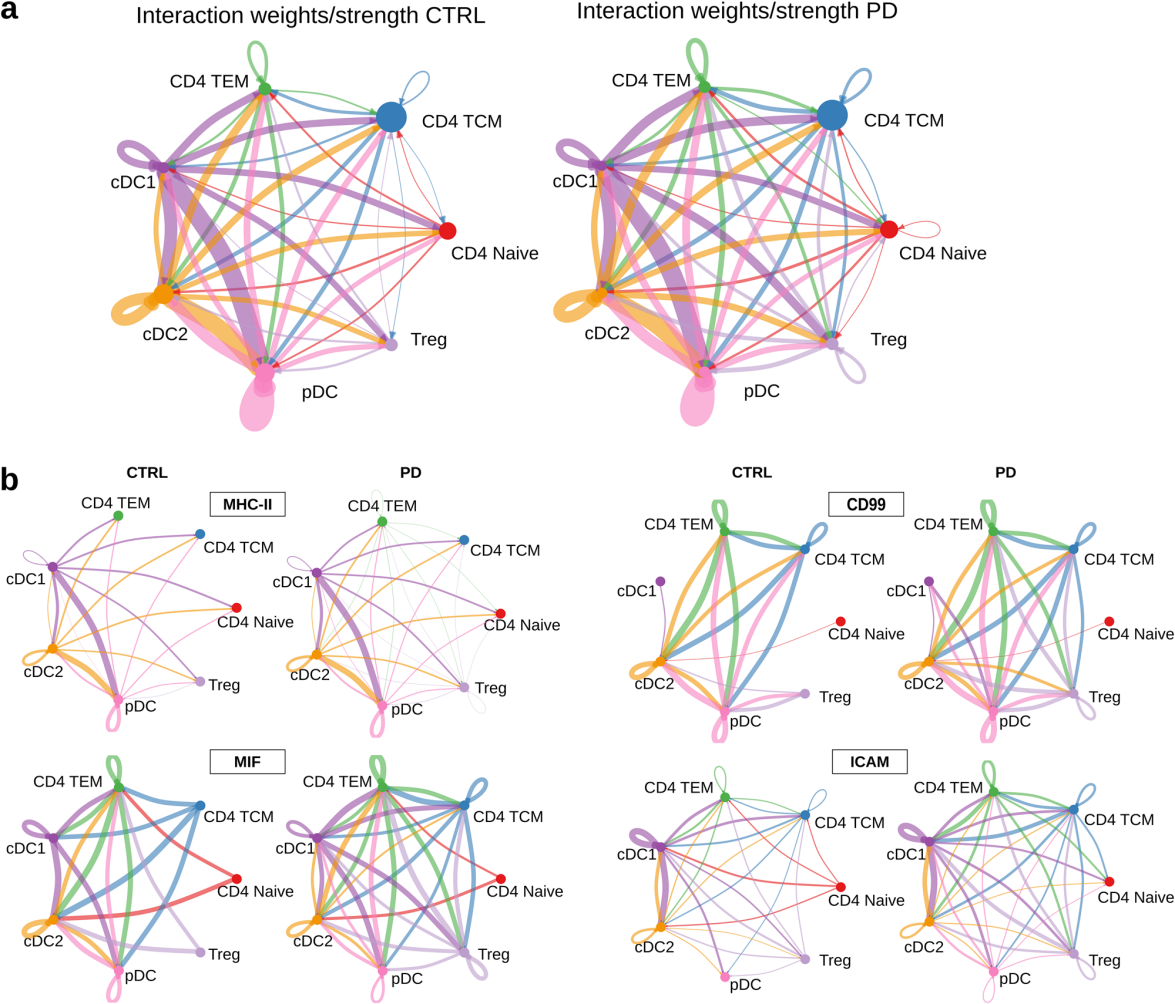
Cell communication networks. **a** Network diagrams illustrate cellular interactions between seven cell subsets in CTRL and PD groups. Edge thickness reflects the overall probability and strength of communication: thicker edges represent higher summed communication probabilities across all significant ligand–receptor pairs. Circle size indicates the number of cells in each subset, with larger circles corresponding to higher cell abundances. **b** Communication networks for MHC-II, MIF, CD99 and ICAM pathways in CTRL and PD cells. PD networks show increased edge density and strength, reflecting enhanced intercellular communication between CD4 T-cells and dendritic cells.

To investigate whether PD reshapes patterns of intercellular signalling between CD4 T-cells and dendritic cells, we used *CellChat*^44^ to model communication networks across conditions. In our dataset, both PD and CTRL cells exhibited a dense and highly connected communication network, indicating that overall CD4-DC cell interactions are preserved in PD (Fig. 8a). Having established this global preservation, we next examined specific signalling pathways relevant to antigen presentation and inflammatory responses, showing increased interaction strengths in PD. The most notable differences were observed in network diagrams for the MHC-II, MIF, CD99 and ICAM signalling pathways, shown in Fig. 8b. In all four subnetworks, the edges generally appear thicker and more numerous for the PD than CTRL interactions with more interconnected nodes, creating a denser communication network. These findings suggest not only a more active but also a more integrated interplay within the immune system in the PD than in CTRL cells, with enhanced bidirectional crosstalk between T-cells and DCs, consistent with a state of heightened immune activation.

### Data validation

This section of the results describes the internal and external validations we performed on our dataset. For an internal validation, we have analysed the following: quality of each sample to identify outliers, per donor overlay in tSNE, biological replicates (samples taken one week apart), the distribution of cells in tSNE for PD cases and controls, and results of differential expression using a pseudobulk approach. The external validation was performed by comparing the gene expression of CD4+ T-cells and DCs from this single-cell study with bulk RNA-seq data (BLUEPRINT project https://projects.ensembl.org/blueprint/) and the reference PMBC single-cell dataset^45^.

We analysed the quality control metrics after cell calling across all samples (Supplementary Data 1) and established that sample S29 should be excluded from our analysis. The fraction of reads in sample S29 was lower than 0.6 (recommended is >0.7), and the estimated number of cells was high (Supplementary Fig. 3a). Furthermore, sample S29 had the lowest mean read and genes per cell called (Supplementary Fig. 3b). The low quality of cells most likely leads to a low mean confidence score for annotating each major cell type (Supplementary Fig. 3c).

**Fig. 9:**
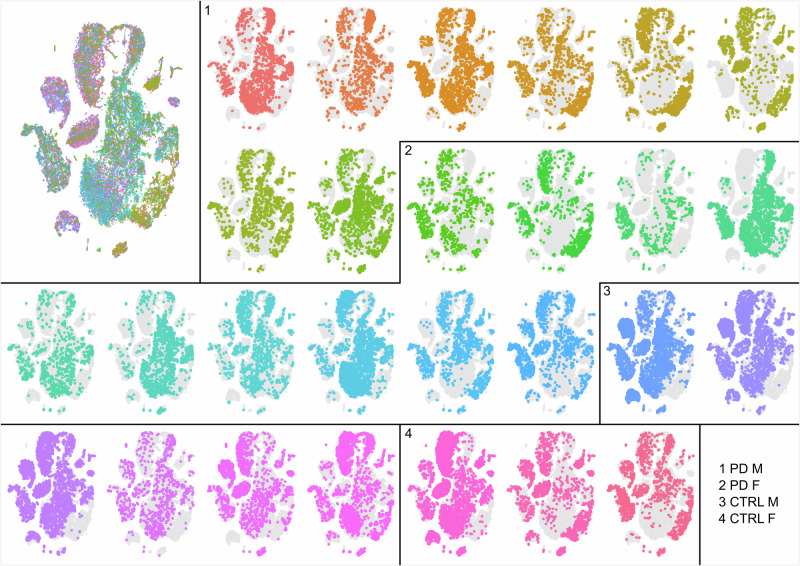
tSNE per donor overlay. Each tSNE plot represents an individual sample. Numbers 1–4 separate samples into groups by condition and sex, as follows: 1-male PD, 2-female PD, 3-male CTRL, 4-female CTRL. PD Parkinson’s disease, CTRL control, M male, F female.

We mapped cells from each donor to ensure an even distribution of cells across different clusters and cell types identified in tSNE (Fig. 9). The variability of cell distribution is consistent and not related to the condition or sex of donors.

Biological replicates of a PD patient were taken one week apart using the same protocol (S28 and S30). DE between these two samples shows a low number of DEGs, suggesting that there is no large variation between the two samples (Supplementary Fig. 4a). Furthermore, there is no obvious separation of these samples observed in tSNE clusters (Supplementary Fig. 4b).

We analysed the distribution of PD and CTRL cells across tSNE for CD4+ T-cells and DCs. Our results show that the cell type clustering is the strongest source of variation in our dataset and that there is no clear separation of cells based on PD and CTRL status (Supplementary Fig. 4c). There is a slight rightward shift of PD cells in the density distribution along the first dimension in both CD4+ T-cells and DCs (Supplementary Fig. 4c).

We applied a pseudobulking approach where we aggregated cells per donor and performed differential expression analysis per cell subtype using a standard bulk RNA-seq pipeline. No genes reached the significance threshold, likely due to loss of power after aggregating. However, fold change values from the pseudobulking approach strongly correlate with fold change values from our single-cell approach; Pearson’s correlation coefficient *r* > 0.8 for all investigated cell subtypes (Supplementary Fig. 5).

External validation was performed by comparing top-expressed genes from our single-cell experiment with bulk RNA-seq (https://projects.ensembl.org/blueprint/) and single-cell PBMC reference dataset^45^. We compared the average gene expression of CD4+ T-cells with bulk RNA-seq from CD4-positive alpha-beta T cells isolated from venous blood and DCs with conventional DCs isolated from cord blood. We found a high correlation of our single-cell gene expression with bulk RNA-seq TPMs for both CD4+ T-cell and DC cell types (Supplementary Fig. 6a, b). Similarly, we compared our data with the average expression of CD4+ T-cells and DCs from the PBMC single-cell data set and found that they highly correlate (Supplementary Fig. 6c, d).

## Discussion

In this study, we provide a comprehensive characterisation of CD4+ T-cell and DC-enriched fractions from PBMCs of PD patients and controls, using single-cell sequencing and transcriptomic analysis. The single-cell sequencing approach on CD4+ T-cell and DC-enriched fractions isolated from PBMCs revealed clusters corresponding to DCs and CD4+ T-cells, along with other cell types, indicating inaccuracies in the magnetic separation method. Notably, most CD4+ T-cells detected in this study expressed *CAMK4* and *BCL11B*—markers not typically used to define this population. Although our findings do not indicate a correlation between this expression pattern and PD, previous studies have linked dysregulation of *BCL11B* and *CAMK4* to PD^46–48^. BCL11B is crucial for T-cell development, while CAMK4 regulates gene expression and immune responses during T-cell activation^49–51^. In this study, however, there is insufficient evidence that these genes exert the PD phenotype, possibly due to limited sample size or methodological constraints. Nevertheless, their role in immune processes could offer valuable insights into immune-related mechanisms underlying PD pathology in future studies.

We further turned our focus to DCs, crucial APCs in CD4+ T-cell activation, subsequently influencing the differentiation of effector cell subtypes. Our flow cytometry analysis revealed significantly diminished levels of circulating DCs in PD patients, especially among mDCs (cDCs), which aligns with earlier findings by Ciaramella et al., who proposed that mDC levels could serve as a potential biomarker for PD. They also observed a correlation between the reduced levels of mDCs and pDCs and the severity of motor symptoms, suggesting potential migration of DCs to affected brain regions^9^. A recent single-cell study on a similarly small dataset to ours, however, shows no DC depletion, so this trend is yet to be validated in a larger cohort of PD patients^52^. In contrast to DCs, our findings did not show significant differences in the overall CD4+ T-cell population or in Th1 and Th17 subsets between PD patients and controls. This does not align with previous studies that reported a decline in circulating CD4+ T-cell numbers^53–55^, as well as a shift toward a pro-inflammatory profile characterised by increased Th1 and Th17 levels or decreased Th2 and Treg subsets in PD patients^53,56^. Such discrepancies may be a result of variations in study cohorts, methodology, or the enhanced resolution provided by scRNA-seq analysis.

Utilising single-cell resolution revealed four CD4+ T-cell subtypes annotated in our samples—naïve CD4+ T-cells, CD4+ TCMs, CD4+ TEMs and regulatory CD4+ T-cells, validated by specific marker gene expression. Differentially-expressed genes between PD patients and controls in the CD4+ TEMs indicated an enrichment for more immune-related terms, including lymphocyte activation and differentiation, compared to other CD4+ T-cell subsets. Previous research emphasised the role of CD4+ T-cells recognising αSyn in autoimmune mechanisms linked to PD, particularly noting stronger αSyn-specific T-cell reactivity in early-stage patients^57^. Although the role of CD4+ TEMs in PD is still underexplored, their ability to migrate to inflamed sites suggests their involvement in neuroinflammatory processes^58^. Elevated CD4+ TEM levels have been associated with cognitive decline, indicating a broader role in cognitive aging^59^. Despite the limited understanding of CD4+ TEMs’ role in PD progression, our findings support their potential role in PD-related immune response and highlight the need for further research into their immunomodulatory potential.

Our analysis also identified three distinct DC subtypes based on specific gene expression markers: cDC1s, cDC2s and pDCs. cDC1s are known to activate CD8 + T-cells and promote Th1 polarisation, while cDC2s primarily interact with CD4+ T-cells and direct their polarisation into various effector phenotypes. In contrast, pDCs are significant producers of type I interferons, essential for antiviral responses^60–62^.

The cDC2 population is increasingly recognised as a heterogeneous group, with previous studies identifying further subdivisions within the subset^40,62^. Villani et al. first identified two distinct cDC2 subsets: DC2 and DC3, marked by different gene expression profiles, with DC3s exhibiting overlapping features of both monocytes and cDC2s^40,61^. Both cDC2 and DC3 subsets contribute to CD4+ naïve T-cell polarisation and inflammation, but DC3s appear particularly relevant due to their dual identity^40,62–64^. In our data, we identified the three DC clusters corresponding to cDC1s, cDC2s and pDCs. We hypothesise that a portion of the cDC2 cluster expressing *CD163* and *CD14* likely represents the DC3 subset. The co-expression of *CD14* and *CD1c* in these presumed DC3s is consistent with the dual phenotype described earlier. Although we did not observe DC3 cells as a separate cluster, likely due to annotation limitations, the presence of these markers suggests their inclusion within cDC2s. This underscores the necessity for refined classification methods in improving immune cell heterogeneity resolution and emphasises the importance of single-cell sequencing in identifying rare immune cell subsets. Importantly, by enriched total DC fractions analysis, we increased our ability to detect rare subtypes that might be missed in bulk PBMC analysis, where DCs comprise only 1–2%^65^. Focusing on enriched DC fractions enabled us to identify a rare population presumed to be DC3 cells, demonstrating the value of this methodology for a detailed analysis of immune cell diversity.

Differential gene expression highlighted cDC2 and CD4+ TEM cells as pivotal drivers of the immune response in PD patients. Our results demonstrated that the DEG profiles of cDC2 and CD4+ TEM subsets were enriched in immune response-related terms, with GSEA revealing shared immune pathways such as ‘T-cell activation’ and ‘positive regulation of immune response’, indicating a functional interplay between them. cDC2s, as APCs, are crucial for priming CD4+ T-cell polarisation and produce T-cell-attracting chemokines like CCL5 and CCL22, along with pro-inflammatory cytokines, enhancing T-cell activation^61,63^. While traditionally linked to Th2 and Th17 polarisation, cDC2s also influence Th1 differentiation depending on the DC subset involved^32^. Leal Rojas et al. identified cDC2s as key promoters of CD4+ TEM responses driven by cytokines such as IL-1β and IL-12p70^64^. The presence of CD4+ helper T-cells in the substantia nigra of PD patients underscores their role in PD pathogenesis^56^. Reactivated CD4+ TEM cells can produce both pro- and anti-inflammatory cytokines, contributing to chronic inflammation in PD^66–68^.

Finally, we investigated CD4+ TEM and cDC2 cells as coordinated mediators of immune responses in PD. Among the top 30 DEGs associated with primary pathways in each cell type, we identified several notably overexpressed genes in PD patients compared to healthy controls. In CD4+ TEMs, genes such as *BCL6*, *ICOS* and *SOCS3* were highly expressed, while in cDC2s, we found increases in *LCK*, *CD3D* and *CD247*. In contrast, cDC2s also exhibited downregulation of *PTGS2* and *SOCS3*. While *ICOS*, *CD3D* and *CD247* have not been extensively studied in the context of PD, *BCL6*—a transcriptional repressor involved in immune response and inflammation—has been linked to neurodegenerative diseases^69^, and its increased expression in our dataset aligns with previous findings in PD^70^. *LCK* has also been implicated in PD, notably promoting Th17 cell differentiation^71,72^. Additionally, *SOCS3* has been associated with neuroinflammation and exacerbation of PD pathology^73,74^. However, we observed cell-type-specific regulation of *SOCS3*, indicating its context-dependent role in immune modulation.

In contrast to our observations, a previous study in mice linked *PTGS2* upregulation in blood to neuroinflammation^75^. This example highlights the essential need for research using human samples and specific cell subtypes to ensure translational relevance to human disease processes.

Moreover, comparative analysis of the PPI networks revealed a highly interconnected structure for cDC2s, indicating a robust framework for immune response orchestration, whereas the CD4+ TEM network was more fragmented, suggesting less specialisation for this disease. Nonetheless, the overlapping pathways between the networks point to T-cell activation as a common biological process in PD, previously described in the literature^76,77^. These insights emphasise the distinct yet complementary roles of these immune subsets in PD pathogenesis, with cell-cell communication networks further revealing heightened crosstalk between CD4+ T-cells and DCs.

This study provides valuable insights into specific immune cell subtypes in PD, yet several limitations should be acknowledged. While cell enrichment effectively identifies rare cell subsets, it may not fully capture the overall immune profile of human blood in PD and could hinder interactions between targeted and non-targeted cells. An additional total PBMC analysis could help address this.

We faced challenges with magnetic separation, affecting sample purity and recovery efficiency, resulting in the loss of some data, as demonstrated by sequencing of CD4+ T-cell and DC-enriched fractions, which identified these cell types, and several others, albeit at lower frequencies. This caveat could be addressed by utilising fluorescence-activated cell sorting for purer populations in future studies. Here, the magnetic separation limitations were addressed by opting for single-cell RNA sequencing to carefully analyse only cells of interest, as opposed to conventional bulk RNA sequencing that averages gene expression across mixed populations of cells. Furthermore, the annotation of CD4+ T-cell subtypes was hindered by reference marker gene sets lacking specificity, causing various CD4+ T-cell populations to cluster together. Incorporating more specific marker genes, such as transcription factors *TBX21*, *GATA3*, *RORC* or cytokines like *IFNG*, *IL4* and *IL17A*, could improve immune heterogeneity resolution^78^. Finally, while our sample size was reasonable and comparable to other studies in the field^27,33,39^, expanding the cohort in future studies would improve the detection of rare cell subsets and better represent inter-patient variability.

In addition, some limitations relate to the availability of detailed pharmacological data, which are often not systematically recorded in routine clinical practice. While all patients received comparable anti-Parkinsonian treatment and were screened to exclude recent infections, vaccinations or biological therapies, subtle effects of unreported medications may have influenced immune profiles. Nevertheless, the consistent clinical background and careful inclusion criteria minimise the potential impact of these factors on our findings. Integrating pharmacological records, standardised cognitive evaluations and α-synuclein biomarkers in future studies will help clarify additional relationships and further refine our understanding of peripheral immune changes in PD.

In conclusion, our study demonstrates the advantages of single-cell RNA sequencing over traditional bulk RNA-seq, particularly in elucidating the complex cellular landscape of PD. This approach enabled the identification of distinct cell types and subtypes, particularly cDC2s and CD4+ TEMs, which may play a pivotal role in PD-related immune responses. We confirmed the robustness of our dataset and revealed critical insights into the immune cell heterogeneity. Furthermore, our pathway network analyses reinforce the significance of the identified DEGs in driving immune mechanisms in PD. Overall, these findings pave the way for future research aimed at targeting specific cellular subpopulations and pathways, enhancing our understanding and potential therapeutic approaches for PD.

## Methods

### Participant selection and sample collection

The study included 17 subjects suffering from Parkinson’s disease (PD) and 10 healthy control subjects without neurological defects, aged 48 to 85 years. The subjects were selected at the University Hospital Centre Zagreb, where the neurology specialists diagnosed patients with PD according to the United Kingdom Brain Bank diagnostic criteria, with the exception that a family history was not considered an exclusion criterion. At the time of enrolment, none of the participants exhibited cognitive symptoms or met diagnostic criteria for Parkinson’s disease dementia or dementia with Lewy bodies. Peripheral blood samples were collected into EDTA-containing blood collection tubes from each participant. One PD participant was sampled twice, a week apart, to provide a biological replicate.

The study was conducted in accordance with the Declaration of Helsinki and received approval from the Ethics Committee of the University Hospital Centre Zagreb, the University of Zagreb School of Medicine and the University Hospital for Infectious Diseases ‘Dr. Fran Mihaljević’. Each participant provided written informed consent before being included in the study.

### PBMC isolation

PBMCs isolation was carried out within 2 to 3 h from the collection, using Ficoll-Paque PLUS (Cytiva Sweden AB, Sweden) density gradient centrifugation. Peripheral blood samples were diluted with phosphate-buffered saline (PBS) in a 1:1 ratio and resuspended to dissociate cell aggregates. The diluted samples were gently layered onto Ficoll in 50 ml tubes and centrifuged. Centrifugation was performed at 400 × *g* for 30 min at room temperature, with the break turned off. After centrifugation, the PBMC ring was transferred into a new tube, washed twice with PBS and centrifuged again at 200 × *g* for 10 min at room temperature. The resulting PBMC pellet was resuspended in RPMI 1640 medium (PAN-Biotech, Germany) and used for the magnetic separation of DCs and CD4+ T-cells. If sufficient blood volume was available, additional PBMC isolation was performed and the pellet was cryopreserved in freezing media containing 90% foetal bovine serum (FBS) and 10% dimethyl sulfoxide for the flow cytometry analysis (Supplementary Table 2). Cells were aliquoted into cryovials, frozen at −80 °C overnight and transferred to liquid nitrogen.

### Magnetic separation of immune cell subpopulations

Using the PBMC suspension, we isolated the CD4+ T-cells and DCs via magnetic separation. CD4+ T cells were separated through positive selection using CD4 MicroBeads (Miltenyi Biotec, Germany) and the MACS separator. The labelled CD4+ T-cells were retained in a magnetic field within the LS column and then eluted with a buffer prepared according to the manufacturer’s instructions. The remaining cells, which did not contain CD4+ T-cells, were used to isolate an enriched population of plasmacytoid and myeloid DCs through negative selection using the human Pan-DC Enrichment kit (Miltenyi Biotec, Germany) to obtain as many DCs as possible. Non-target cells labelled with lineage-specific monoclonal antibodies were retained within the LS column, allowing the DCs to pass through and be collected in a new tube.

The cell count of CD4+ T-cells and DCs was determined using a haemocytometer, and the concentration of each cell type was calculated for each sample. The concentration of CD4+ T-cells was then adjusted to match the concentration of DCs at 500 cells/µl to enable equal comparison and analysis of gene expression between cell subtypes. Finally, the cell suspensions were mixed in equal parts to create samples for pre-library preparation for scRNA-seq.

### Flow cytometry

Before analysis, PBMCs were thawed in warm RPMI medium, washed with PBS + 2% FBS and counted. For the analysis of the DCs, 2 × 10⁶ of PBMCs were stained with FITC lineage markers (lin), PerCP-HLA-DR, APC-CD123, Pacific Blue-CD11c and viability dye Live/Dead near IR. For Th1 and Th17 analysis, 1 × 10⁶ cells were stained with FITC-CD3, PE-CD8, PerCP-Cy5.5-CD194, APC-CCR10, Pacific Blue-CD183, BV605-CD196 and Live/Dead near IR. All antibodies were obtained from BioLegend (San Diego, USA), and the viability dye from Thermo Fisher Scientific (Waltham, USA). After 20 min at 10 °C, cells were washed with PBS + 2% FBS and fixed with 2% paraformaldehyde. Compensation beads were used to generate the compensation matrix, and fluorescence minus one (FMO) controls were used to set gates and thresholds for positive staining. Flow cytometry was performed using a Cytoflex S analyzer (Beckman Coulter, USA). Data were acquired using CyteExpert software (Beckman Coulter, USA). Compensation and analysis were done with FlowJo™ v10.10. Software (BD Life Sciences, USA). Myeloid dendritic cells (mDCs) and plasmacytoid dendritic cells (pDCs) were identified using a previously established gating strategy^79^. Briefly, cells were first gated on the leucocyte population based on forward scatter (FSC-H) and side scatter (SSC-H) properties. Doublets were excluded by gating on SSC-A vs. SSC-H. Live cells were selected using a viability dye. Dendritic cells were identified as lineage-negative (Lin−) cells, followed by gating on HLA-DR+ events. mDCs and pDCs were further distinguished based on their expression of CD11c and CD123, with mDCs defined as CD11c^+^CD123^−^ and pDCs as CD11c^−^CD123^+^. Gating strategy for dendritic cell identification is shown in Supplementary Fig. 7.

CD4+ T-cell subsets were identified from PBMCs. Debris and doublets were excluded, and live lymphocytes were selected based on FSC and SSC, with dead cells removed using a viability dye. CD4+ T cells were gated within the CD3+ population, and Th1 and Th17 subsets were distinguished by chemokine receptor expression. Th1 cells were defined as CD183+ (CXCR3+) CD194− (CCR4−), while Th17 cells were CD194+ (CCR4+) CD183− (CXCR3−), with CD196 (CCR6) used to confirm the Th17 phenotype. The gating strategy followed Mahnke et al.^80^.

Statistical analysis was performed using R (version 4.4.1). The normality of data distribution was assessed using the Shapiro–Wilk test. For normally distributed variables, comparisons between groups were made using Student’s *t* test, while the Wilcoxon rank-sum test was applied to non-normally distributed variables. Data were visualised using boxplots with individual data points to represent the data distribution and group medians. *P* values below 0.05 were considered statistically significant.

### Single-cell RNA sequencing

We constructed the gene expression (GEX) libraries using the Chromium Next GEM Single Cell 5’ Reagent Kit v2 (dual index chemistry) (10× Genomics, USA) following the instructions (document CG000331 revE). Briefly, the cell suspension was first diluted in nuclease-free water to acquire the target cell count of 5000 (10,000 for the initial batch). After the addition to the master mix, the cell suspension was loaded on the microfluidic Next GEM Chip K together with Next GEM Single Cell 5’ gel beads v2 and partitioning oil and run on the Chromium X instrument (10× Genomics, USA). This instrument enabled the formation of gel beads in emulsion (GEMs), and the single cells’ mRNAs were uniquely barcoded. RNA transcripts were reverse-transcribed within the created droplets, and subsequently, the barcoded cDNA products were pooled. Pooled cDNA further underwent purification using Dynabeads MyOne SILANE (ThermoFisher Scientific, USA) and SPRIselect beads (Beckman Coulter, USA), and amplified through polymerase chain reaction (PCR) in 14 cycles. Quality control (QC) and quantification of amplified cDNA were performed on an Agilent Bioanalyzer high-sensitivity chip, and run on Bioanalyzer 2100 (Agilent Technologies, USA). Before constructing the 5’ GEX libraries, cDNA yield was calculated for each sample following the manufacturer’s guidelines.

After the 5’ GEX library construction, the final QC of the libraries was performed on the Bioanalyzer 2100. Libraries were then pooled four samples at a time, denatured, and diluted to a loading concentration of 1.5 pM using the standard normalisation method following the Illumina guidelines (Document #15048776 v09, protocol A). Prepared libraries were sequenced using the High Output 150 cycles sequencing kit and Illumina NextSeq 550 platform (Illumina, USA), with a sequencing depth of 20,000 reads per cell.

### Alignment and quality control of sequencing data

We used the Cell Ranger v7.2 (10x Genomics, USA) tool and standard pipeline for processing scRNA-seq data. The sequencing data were first demultiplexed by concatenating FASTQ files per sample. The *count* function from the Cell Ranger v7.2 tool performs multiple steps, including read alignment, gene expression quantification and individual cell calling. Alignment and feature counting were performed against the prebuilt human genome reference (GRCh38, 2020) downloaded from the 10x Genomics website (https://cf.10xgenomics.com/supp/cell-exp/refdata-gex-GRCh38-2020-A.tar.gz). The cell calling step identifies droplets that do not contain cells and filters them out of the raw count matrix. Quality control metrics from this step are summarised in the Supplementary Data 1.

### Single-cell data pre-processing

Most of the subsequent analysis was performed using R v4.2 programming language and Seurat package v5.0 executed in a Singularity container to ensure reproducibility. The image used is available on Docker Hub (https://hub.docker.com/r/prskalok/seurat). During quality control, we performed several filtering steps: (i) filtered out cells that contained more than 5% of mitochondrial genes, (ii) excluded cells which expressed less than 200 genes or less than the upper threshold, which was defined as 3 median absolute deviations from the median of a sample, (iii) filtered out cells that had >20,000 unique molecular identifiers (UMIs). We normalised using logNormalize, then we used the vst method to identify the top 2000 highly variable features. Next, we scaled the data and performed principal component analysis to reduce the dimensionality of the data, which we used in downstream analysis. We used t-distributed Stochastic Neighbour Embedding (tSNE) for visualisation. Doublet identification was performed using DoubletFinder v2.0.4. We performed a parameter sweep, and for most samples we used the default optimal parameters. However, for assessing the neighbourhood size (pK), we followed the protocol suggested by the authors of the tool^81^. We performed sample integration with reciprocal principal component analysis (RPCA) followed by shared nearest neighbour (sNN) clustering.

### Cell type annotation

For cell type annotation, we applied a reference-based approach using a PBMC data set^45^. This approach utilises anchors for harmonising data sets, followed by projecting reference labels onto the query data set. The labels that are included in the reference databases are categorised in two levels: broad cell types (e.g. DC, CD4+ T and CD8+ T) and subtypes (e.g. pDC, cDC1 and cDC2). Final cell type clusters were defined by combining sNN clustering and reference-based cell type annotation.

Trajectory and pseudotime analysis were performed using Monocle v2.3^82^ on CD4 and DC cells. Due to a large number of annotated CD4+ T-cells, a random sample of 5000 cells was selected for calculating the trajectory. All DC cells were used for calculating the trajectory. The top 500 highly variable genes were used as the ordering filter.

### Differential expression analysis

Differential expression (DE) was performed with the Seurat function FindMarkers using the Wilcoxon Rank Sum test. Raw *p* values were adjusted with Bonferroni correction for multiple testing, hereafter reported as *p*_adj_. We set the significance threshold for DE genes as *p*_adj_ < 0.05 and absolute log2 fold change 0.5. The pathway enrichment was performed on a local version of R v4.2 and gProfiler2 v0.2. We used annotated human genes as a background set for the hypergeometric test, and the Set Counts and Sizes correction for multiple testing. Visualisations were generated using ggplot2 v3.4 and GOplot v1.0.

The STRING database^83^ was used to predict potential functional interactions between differentially expressed genes (DEGs) across cell subtypes. The STRING tool specifically identified potential interactions among DEGs related to different cell types, leveraging active interaction sources such as experimental repositories, computational prediction methods, and public text collections. The analysis was limited to the species *Homo sapiens* and considered interactions with a combined score greater than 0.4. To visualise the interaction network, we utilised Cytoscape software (version 3.10.3)^84^. To identify highly connected regions (functional clusters) within the STRING networks, we employed the ClueGO Cytoscape plugin (v2.5.10)^85^, applying the following criteria: GO tree levels 3–8, a minimum of 3 genes per term, and a minimum of 4% of associated genes. Right-sided hypergeometric test was used with Bonferroni correction for multiple testing. Term fusion and grouping were enabled, and the Kappa score threshold was set at 0.4.

To account for intercellular communication differences between PD and CTRL cells, we utilised CellChat v2.1^44^. This tool quantitatively infers and compares intercellular signalling based on the average expression of ligand-receptor pairs across cell populations.

### Data validation

Pseudobulking approach was implemented by aggregating gene counts of cells per donor and per cell subtype. This resulted in a gene sample matrix equivalent to a bulk-RNA seq dataset, which was further analysed with the standard DESeq2 pipeline^86^. Log2 Fold Change values were extracted for genes that achieved statistical significance using the single-cell approach and FindMarkers function.

External data validation was performed by comparing top-expressed genes using a bulk RNA-seq data set downloaded from the Blueprint project (https://projects.ensembl.org/blueprint/) and a single-cell reference data set of PBMCs^45^. From the Blueprint project, we used six samples of CD4-positive alpha-beta T cell data from venous blood and three samples of conventional dendritic cells from cord blood. We calculated the mean of TPMs across the samples. Gene expression data from the reference single-cell data set^45^, as well as our data set, were calculated as mean normalised expression across cells annotated as CD4 or DC. Validation was performed by comparing the top 500 expressed genes from our data set using Spearman correlation.

## Supplementary information

Supplementary Information

Supplementary Data 1

Supplementary Data 2

Supplementary Data 3

## Data Availability

The single-cell RNA sequencing data obtained during this study will be made publicly available in the GitHub repository [https://github.com/PKatarina/PD_SingleCell] at the publication date of this article. All other data supporting this study are available from the corresponding authors upon reasonable request.
