# Supplementary material for: Single-cell analysis of the peripheral immune landscape in Parkinson’s disease: insights into dendritic cell and CD4+ T-cell transcriptomics
Source: NPJ Parkinsons Dis. 2026 Feb 11;12:73. doi: 10.1038/s41531-026-01283-1 (PMC13004879; doi:10.1038/s41531-026-01283-1)
Supplement: Supplementary file 1 — Supplementary Information [file 41531_2026_1283_MOESM1_ESM.docx]

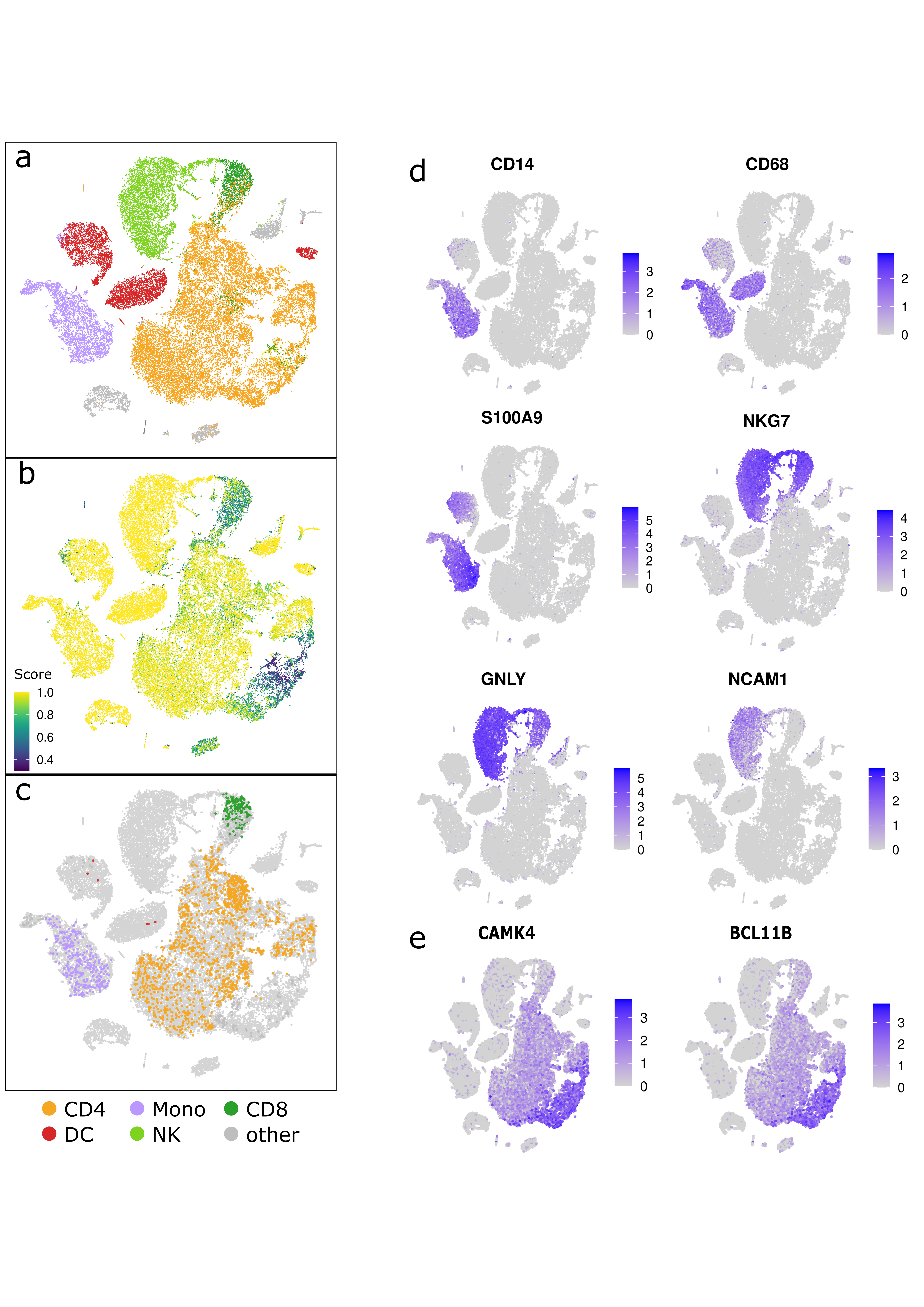


**Supplementary Fig. 1** Extended cell type annotation. **a** Unrefined cell type annotation using reference-based annotation. **b** Annotation confidence score indicating low confidence for a set of CD4+ T-cells later excluded from the analysis. **c** tSNE highlighting the cells corresponding to sample S13, consisting exclusively of the CD4 fraction. **d** Feature plots displaying the expression of key marker genes for monocytes (*CD14*, *CD68*, *S100A9*) and NK cells (*NKG7*, *GNLY*, *NCAM1*). **e** Feature plots displaying the expression of *CAMK4* and *BCL11B* in the majority of CD4+ T-cells. Higher expression levels are indicated in purple.


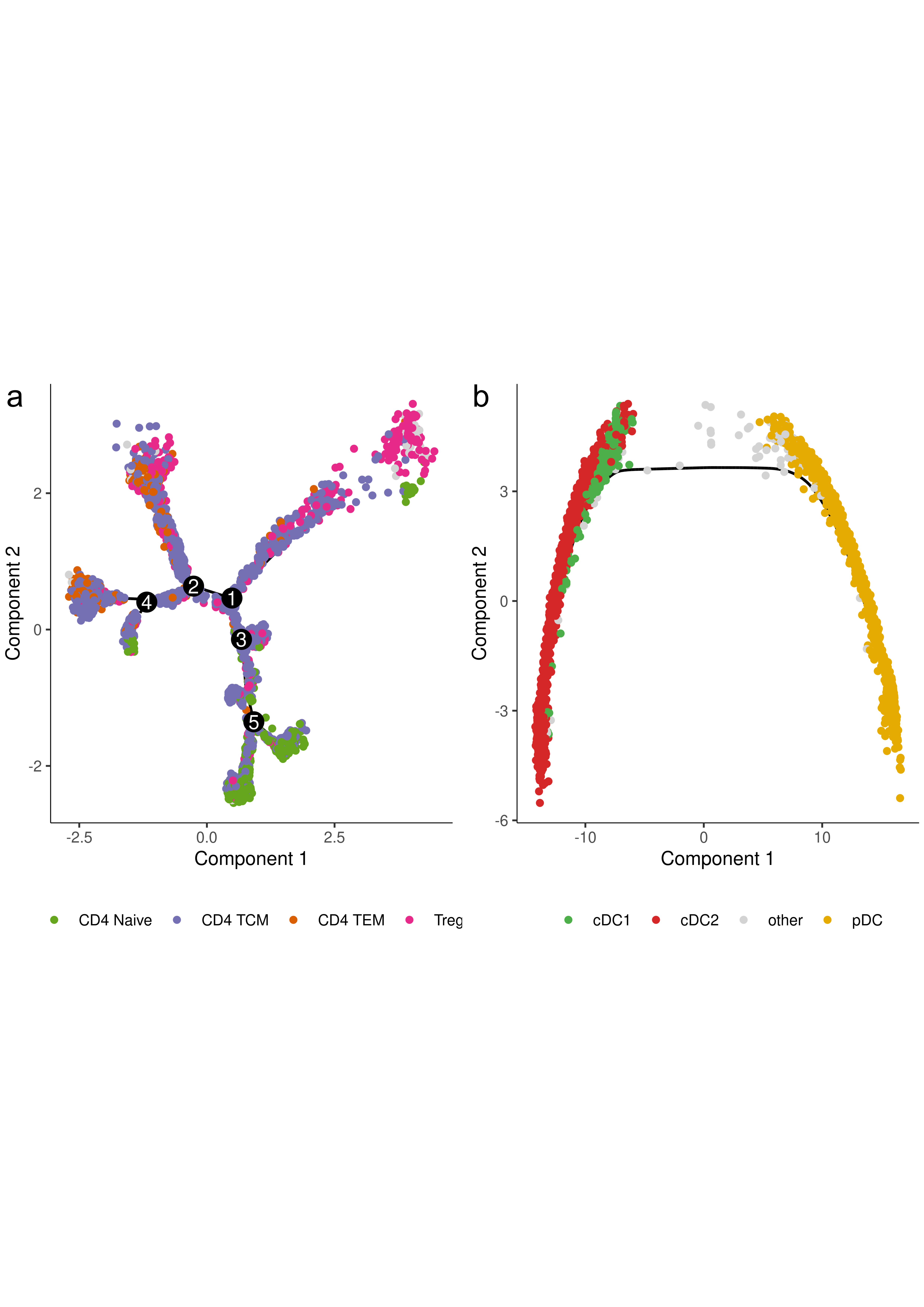
**Supplementary Fig. 2** Differentiation trajectory trees for **a** CD4+ T-cells and **b** Dendritic cells (DCs). cDCs and pDCs don’t derive from a shared progenitor, and thus do not form a branching trajectory.

**
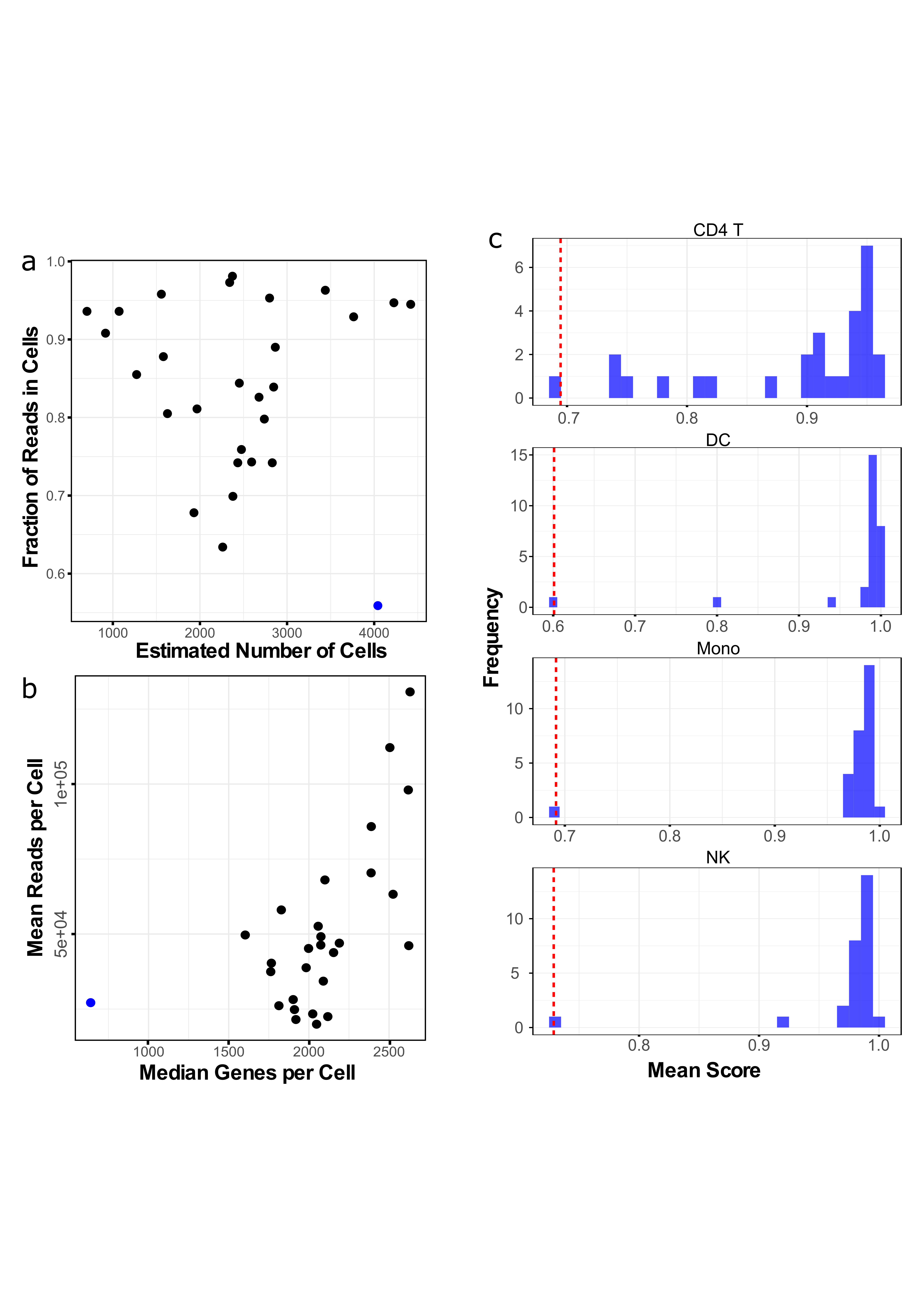
**

**Supplementary Fig. 3** Exclusion criteria for sample S29. **a** Scatter plot illustrates sample S29 (blue dot) exhibiting the lowest fraction of reads detected in cells, together with a very high estimated number of cells. **b** Sample S29 shows very low mean reads per cell and a low median number of detected genes per cell. **c** Using reference-based annotation, the average mean prediction confidence score was calculated for each major cell type (CD4 T, DC, Mono, and NK) across all samples. The confidence score for sample S29 (red line) was the lowest across all cell types.


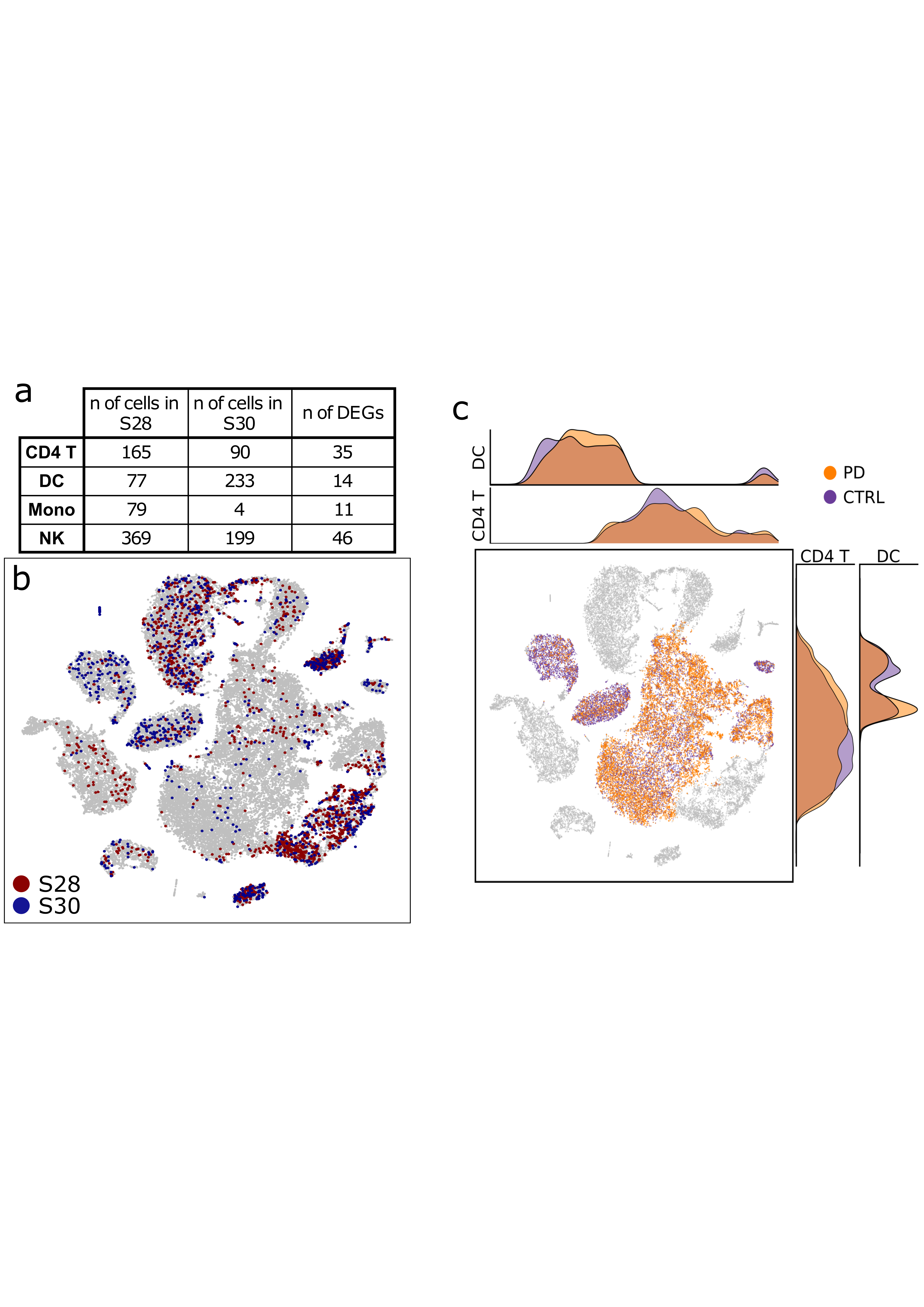


**Supplementary Fig. 4** Biological replicates and PD cell distribution. **a** Number of cells detected in biological replicates taken one week apart (S28 and S30), along with the number of DEGs obtained when comparing them. **b** Cell distribution plot corresponding to biological replicates S28 and S30. **c** tSNE showing cells corresponding to PD and CTRL samples, with the density distribution of these cells across CD4 and DC cell types.


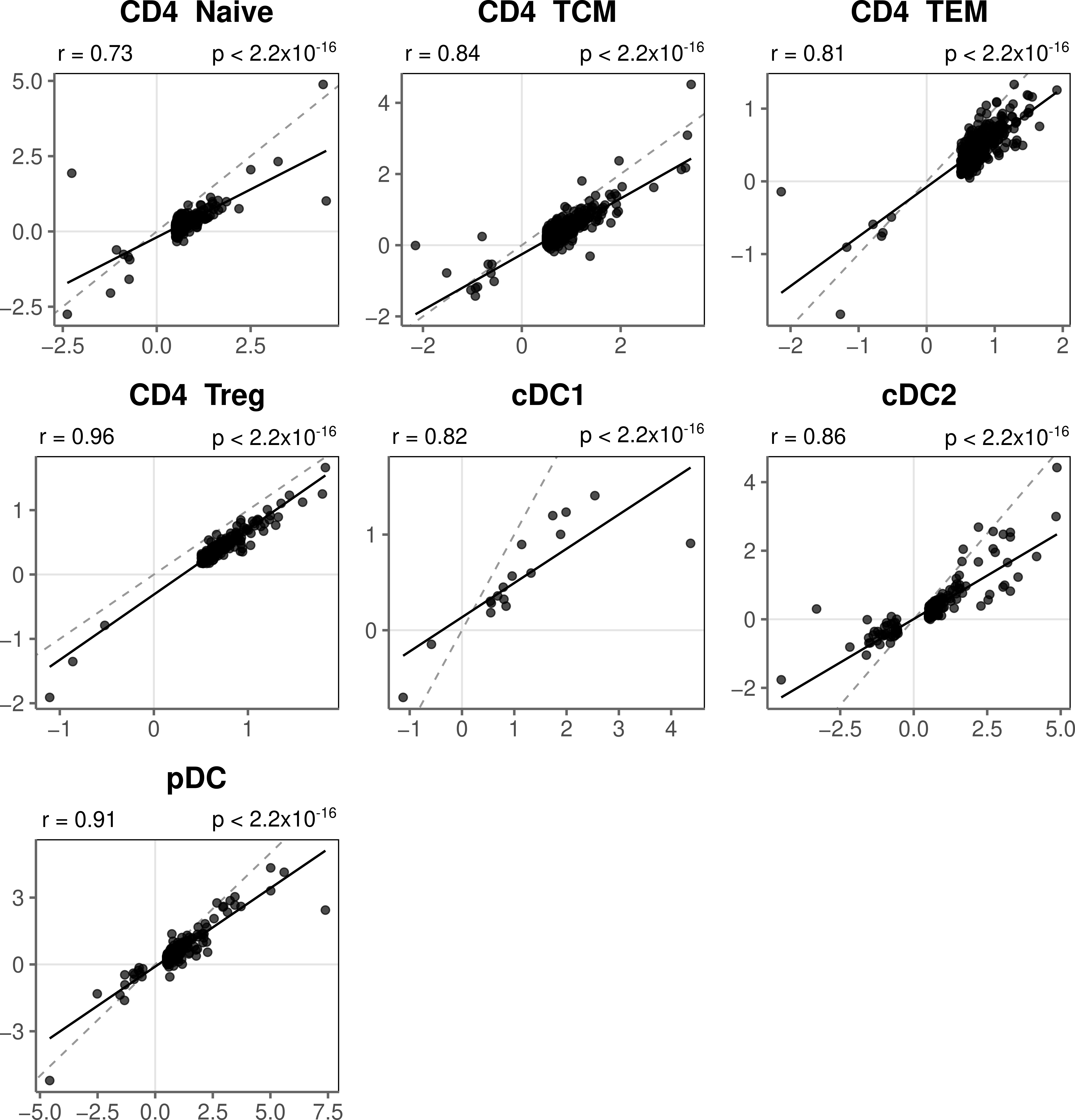


**Supplementary Fig. 5** Scatterplots showing linear relationship between log_2_ (Fold Change) values calculated with single-cell approach (x-axis) and pseudobulking approach (y-axis). Only genes achieving the significance threshold between PD and CTRL from the single cell approach (Bonferroni p_adj_ < 0.05 and |log_2_FC| > 0.5, FindMarkers function) are used for calculation. The log_2_FC values from the pseudobulking approach were calculated using a standard DESeq2 pipeline.


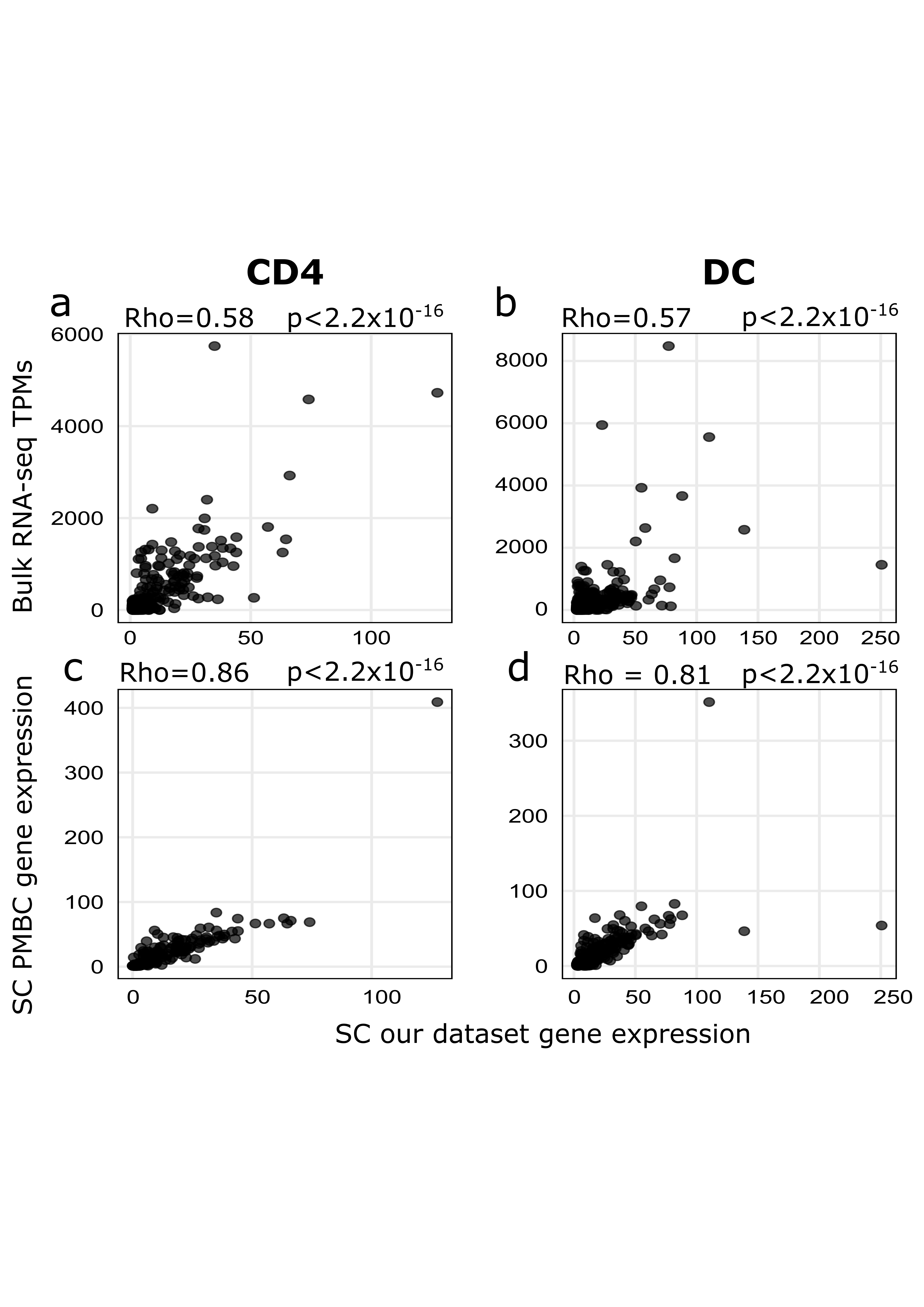


**Supplementary Fig. 6** Scatter plots showing relationship between gene expression in our data set and bulk RNA-seq and single-cell (SC) PBMC data across CD4 and DC cell types. Spearman correlation (expressed as Rho) was calculated for each comparison. Panels display comparison of our data set with: **a** Bulk RNA-seq in CD4, **b** Bulk RNA-seq in DC, **c** SC PBMC in CD4 and **d** SC PBMC in DC. Statistical significance was determined at p<0.05.


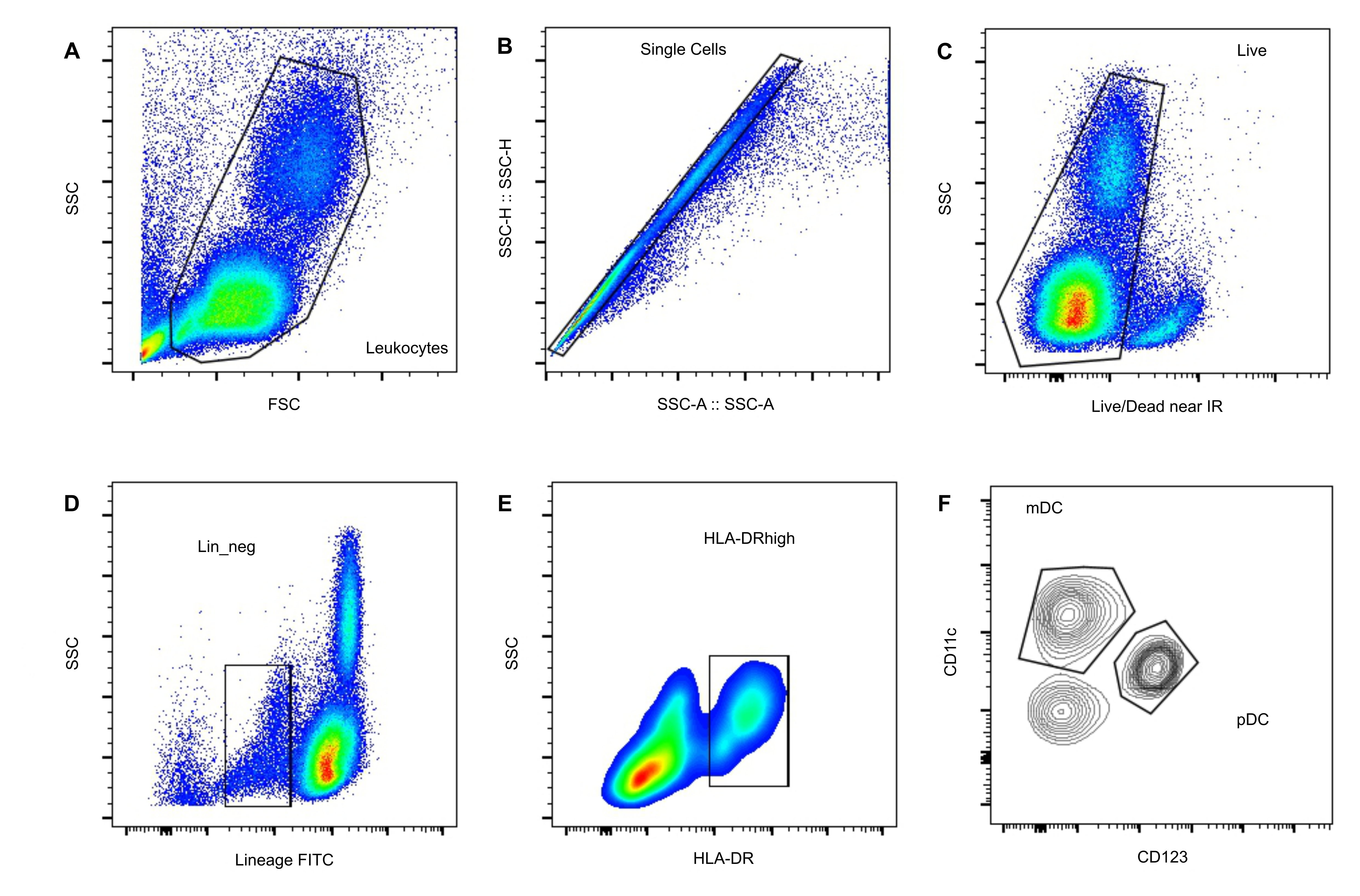


**Supplementary Fig. 7** Gating strategy for dendritic cell identification. Leukocytes were gated based on FSC/SSC characteristics (**A**), followed by exclusion of doublets (**B**) and dead cells (**C**). Lineage-negative (Lin_neg) HLA-DR^high^ cells were selected (**D–E**), and myeloid (mDC) and plasmacytoid (pDC) subsets were distinguished by CD11c and CD123 expression (**F**).

**Supplementary Table 1 -** Wilcoxon Rank Sum test p-values for differences in the proportions of CD4+ T-cell and DC subtypes between PD and CTRL groups. No significant differences (p < 0.05) were detected for any subtype.

| **Cell type** | **Cell subtype** | **Median PD** | **Median CTRL** | **Wilcoxon p-value** |
| --- | --- | --- | --- | --- |
| CD4 T | CD4 Naive | 0,1575492341 | 0,1540229885 | 0,979888944 |
| CD4 T | CD4 TCM | 0,6294326241 | 0,6186868687 | 0,595850709 |
| CD4 T | CD4 TEM | 0,0763621223 | 0,0804597701 | 1 |
| CD4 T | Treg | 0,1022222222 | 0,1002031144 | 0,899684541 |
| DC | cDC1 | 0,0643776824 | 0,0922619048 | 0,090841735 |
| DC | cDC2 | 0,402173913 | 0,4838709677 | 0,561167955 |
| DC | pDC | 0,5556287031 | 0,4623655914 | 0,240923013 |

**Supplementary Table 2** – List of all the samples included in scRNA-seq and flow cytometry analysis.

| **#** | **Sample** | **Status** | **Sample included in scRNA-seq** | **Sample included in Flow Cytometry** |
| --- | --- | --- | --- | --- |
| 1 | **S01** | CTRL | Yes | Yes |
| 2 | **S02** | CTRL | Yes | Yes |
| 3 | **S03** | CTRL | Yes | Yes |
| 4 | **S04** | CTRL | Yes | Yes |
| 5 | **S05** | PD | Yes | Yes |
| 6 | **S06** | PD | Yes | Yes |
| 7 | **S07** | PD | Yes | No |
| 8 | **S08** | PD | Yes | Yes |
| 9 | **S09** | PD | Yes | Yes |
| 10 | **S10** | PD | Yes | Yes |
| 11 | **S11** | PD | Yes | No |
| 12 | **S12** | CTRL | Yes | Yes |
| 13 | **S13** | PD | Yes | No |
| 14 | **S14** | PD | Yes | No |
| 15 | **S15** | CTRL | Yes | No |
| 16 | **S16** | PD | Yes | No |
| 17 | **S21** | PD | Yes | Yes |
| 18 | **S22** | CTRL | Yes | Yes |
| 19 | **S23** | CTRL | Yes | Yes |
| 20 | **S24** | PD | Yes | Yes |
| 21 | **S25** | PD | Yes | Yes |
| 22 | **S26** | CTRL | Yes | Yes |
| 23 | **S27** | PD | Yes | Yes |
| 24 | **S28*** | PD | Yes | Yes |
| 25 | **S29** | CTRL | Yes | Yes |
| 26 | **S30*** | PD | Yes | No |
| 27 | **S31** | PD | Yes | Yes |
| 28 | **S32** | PD | Yes | Yes |

*replicates, samples collected 1 week apart

**Supplementary Data 1** – Quality control metrics summary of all samples.

**Supplementary Data 2** - Complete list of differentially expressed genes in analysed cell subsets.

**Supplementary Data 3** – GSEA analysis - full set of terms and results.
